# The operational model of allosteric modulation of pharmacological agonism

**DOI:** 10.1038/s41598-020-71228-y

**Published:** 2020-09-02

**Authors:** Jan Jakubík, Alena Randáková, Nikolai Chetverikov, Esam E. El-Fakahany, Vladimír Doležal

**Affiliations:** 1 Institute of Physiology CAS, Vídeňská 1083, 142 20 Prague, Czech Republic; 2 Department of Experimental and Clinical Pharmacology, University of Minnesota College of Pharmacy, Minneapolis, MN 55455 USA

**Keywords:** Pharmacodynamics, Receptor pharmacology

## Abstract

Proper determination of agonist efficacy is indispensable in the evaluation of agonist selectivity and bias to activation of specific signalling pathways. The operational model (OM) of pharmacological agonism is a useful means for achieving this goal. Allosteric ligands bind to receptors at sites that are distinct from those of endogenous agonists that interact with the orthosteric domain on the receptor. An allosteric modulator and an orthosteric agonist bind simultaneously to the receptor to form a ternary complex, where the allosteric modulator affects the binding affinity and operational efficacy of the agonist. Allosteric modulators are an intensively studied group of receptor ligands because of their selectivity and preservation of physiological space–time pattern of the signals they modulate. We analysed the operational model of allosterically-modulated agonism (OMAM) including modulation by allosteric agonists. Similar to OM, several parameters of OMAM are inter-dependent. We derived equations describing mutual relationships among parameters of the functional response and OMAM. We present a workflow for the robust fitting of OMAM to experimental data using derived equations.

## Introduction

Monod et al.^1^ originally introduced the concept of allosterism. Since then the concept of allosterism extended to many various fields of research spanning from DNA expression via metabolism to ion channels and G-protein coupled receptors^2, 3^. Allosteric ligands bind to a site that is distinct from the orthosteric site on a receptor. An orthosteric and allosteric ligand can bind to the receptor concurrently and form a ternary complex where they reciprocally modulate the binding affinity of each other. Moreover, the binding of an allosteric modulator may also affect the efficacy of an orthosteric agonist in eliciting a functional response.

As allosteric binding sites need not accommodate natural agonist they are subject to less evolutionary pressure. This leads to a less-conserved structure of an allosteric binding site^4^. The evolutionary adaptation mechanisms may even help maintain, optimize or regulate allosteric behaviour of signalling macromolecules^5^. Thus, higher binding selectivity can be achieved for allosteric than orthosteric ligands. Even if an allosteric site is conserved, selectivity can be achieved via optimization of cooperativity with the orthosteric ligand^6^. An additional advantage of allosteric modulators is the conservation of the space–time pattern of signalling, as their action is restricted to modulation of signalling mediated by the intermittent quantum release of a neurotransmitter and where receptors responsive to the neurotransmitter are expressed^7^. These special characteristics of allosteric receptor modulators have stimulated intensive studies towards their application in therapy of a variety of disorders^8–10^. However, the nature of allostery makes the task challenging^11,12^.

In pharmacological terms, efficacy is the ability of an agonist to induce a maximal functional response in a cell, tissue or organ. The response to the agonist may vary among systems. Thus, absolute quantification of efficacy is impossible. In 1983, Black and Leff presented a model, termed the operational model (OM) of pharmacological agonism^13^. The OM calculates a parameter τ_A_ termed “operational efficacy” of agonist from two “objective” parameters, the equilibrium dissociation constant of agonist (K_A_) at the active state of the receptor and the maximal response of the system (E_MAX_)^14^. It has been shown that these three parameters (E_MAX,_ K_A_, and τ_A_) are inter-dependent and, therefore, we proposed a two-step procedure to overcome this pitfall^15^.

**Figure 1:**
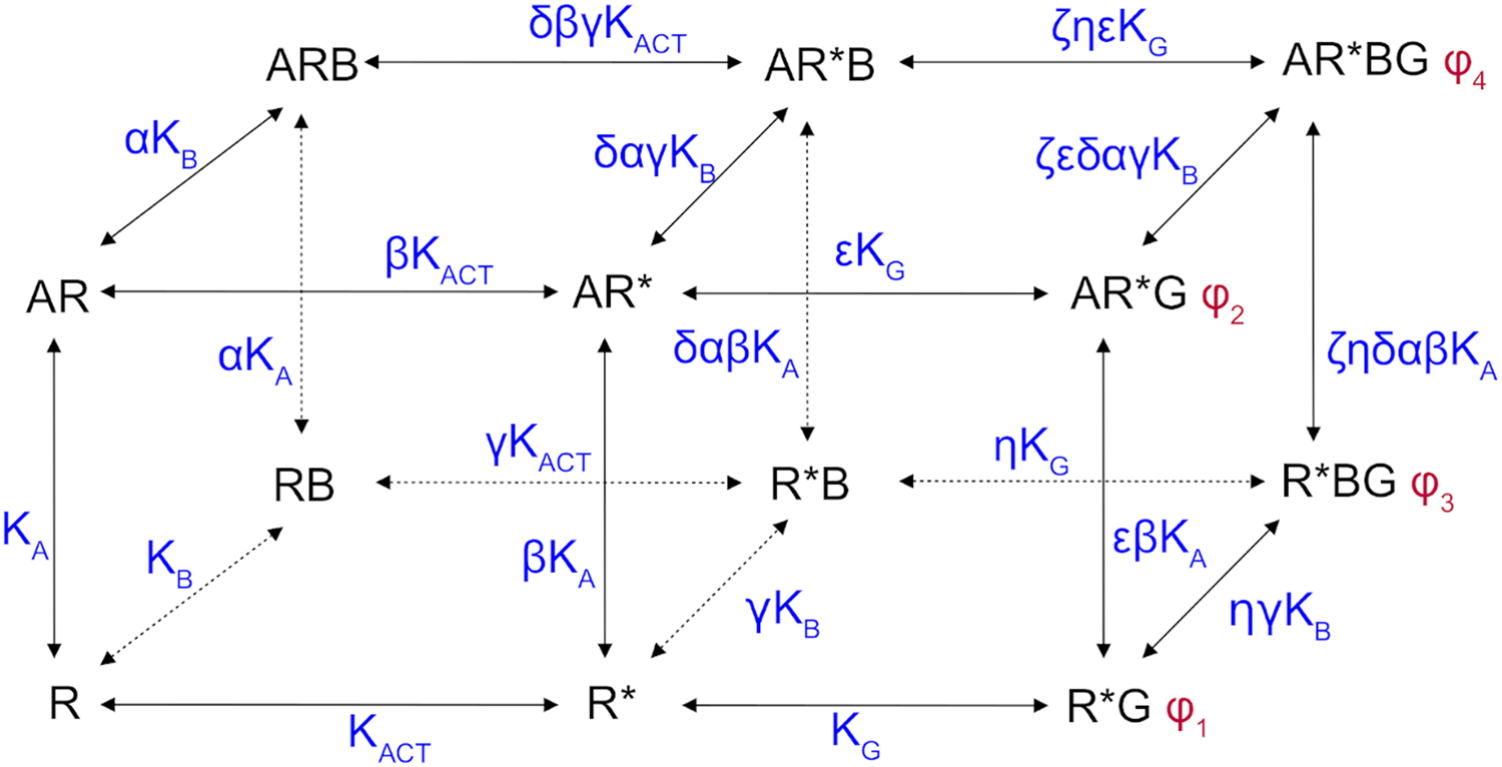
The cubic ternary complex model of allosteric modulation of receptor activation. Receptor exists in two states; inactive, R, and active, R*. The equilibrium between these two states is given by the activation constant K_ACT_. An orthosteric agonist A binds to the inactive receptor with an equilibrium dissociation constant K_A_. An allosteric modulator binds to the inactive receptor with an equilibrium dissociation constant K_B_. α, the factor of binding cooperativity between the orthosteric agonist A and allosteric modulator B. β, the factor of cooperativity between binding of agonist A and receptor activation. γ, the factor of cooperativity between the binding of allosteric modulator B and receptor activation. δ, the factor of cooperativity between the binding of an allosteric modulator and agonist-induced receptor activation. Further, equilibrium dissociation constant K_G_ of G-protein, G, may differ among individual complexes of R* by cooperativity factors ε and η. Also signalling efficacy φ of G-protein may differ among complexes. Adopted from Weiss et al. 1996.

**Figure 2:**
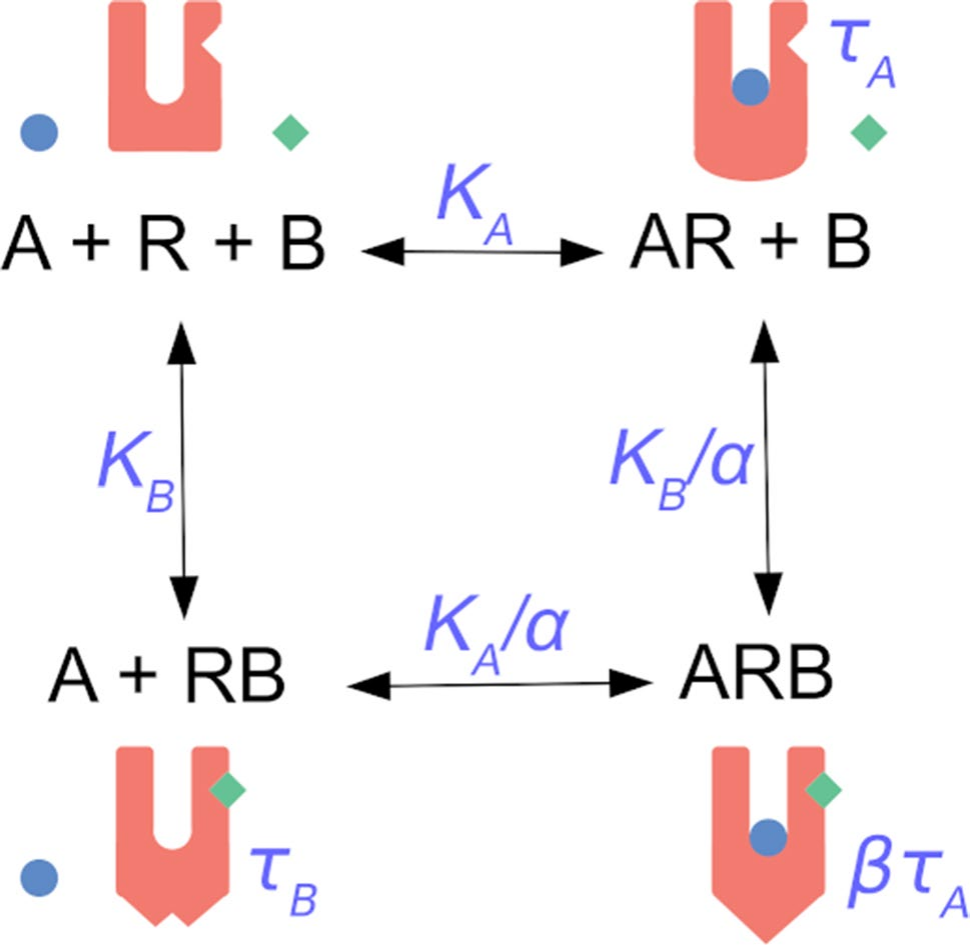
Scheme of the ternary complex model of allosteric interaction. Representation of allosteric interaction between an orthosteric agonist A (blue circle) and an allosteric modulator B (green diamond) at the receptor R (red U-shape). An orthosteric agonist A binds to the receptor R with an equilibrium dissociation constant K_A_. An allosteric modulator B binds to the receptor R with an equilibrium dissociation constant K_B_. The orthosteric agonist A and the allosteric modulator B can bind concurrently to the receptor R to form a ternary complex ARB. The factor of binding cooperativity α is the ratio of the equilibrium dissociation constant to empty receptor to the equilibrium dissociation to binary complex AR or RB. The complex of receptor and orthosteric agonist (AR) has operational efficacy τ_A_. The complex of receptor and allosteric agonist (RB) has operational efficacy τ_B_. The factor of operational cooperativity β is the ratio of operational efficacy of the ternary complex ARB to operational efficacy of binary complex AR.

The thermodynamically complete description of allosteric modulation of receptor activation is described by a cubic ternary complex (CTC) model (Fig. 1)^16^. However, such a heuristic model is not suitable for experiment analysis, as its parameters are next to impossible to estimate. Parsimonious models suit experiment analysis better. In case of agonist binding to the receptor that is allosterically modulated, the parsimonious OM needs to be extended by the operational factor of cooperativity (β) and equilibrium dissociation constant of the allosteric modulator (K_B_) at the active state of the receptor (Fig. 2)^17^. The operational factor of cooperativity, β, quantifies the overall effect of an allosteric modulator on operational efficacy of an orthosteric agonist, τ_A_, and thus brings inter-dependence of three OM parameters with K_B_. The resulting operational model of allosterically-modulated agonism (OMAM) is thus very complex and has five inter-dependent parameters: E_MAX,_ K_A_, τ_A_, K_B_ and β.

Several allosteric ligands of various receptors activate the receptor in the absence of an agonist^18–24^. These ligands are termed allosteric agonists. Their intrinsic activity, τ_B_, can be ranked according to the OM. The OMAM that describes the functional response to an agonist in the presence of an allosteric agonist is even more complex than the OMAM for pure allosteric modulators that lack agonistic activity. The number of inter-dependent parameters rises to six.

In this paper, we analyse the OMAM and derive equations describing mutual relations among parameters of functional response and OMAM, both for pure allosteric modulators and allosteric agonists. These equations would be useful in the analysis of experimental data of such complex systems. For this purpose, we also present a workflow for the reliable fitting of OMAM to experimental data using the derived equations to avoid fitting equations with inter-dependent parameters.

## Description and analysis of models

### The operational model of agonism

The pharmacological response to an agonist depends on the properties of the agonist and the system in which the response is measured. The operational model (OM) of agonism describes the system response using three objective parameters^13^. According to OM the response of the system follows Eq. (1).

1 $$Response = \frac{{\left[ A \right]\tau_{A} E_{MAX} }}{{\left[ A \right]\left( {\tau_{A} + 1} \right) + K_{A} }}$$

where [A] is the concentration of an agonist, E_MAX_ is the maximal possible response of the system, K_A_ is the equilibrium dissociation constant of the agonist-receptor complex and τ_A_ is the operational factor of efficacy. According to the OM, EC_50_ is related to K_A_ according to the following Eq. (2).

2 $$EC_{50} = \frac{{K_{A} }}{{\tau_{A} + 1}}$$

The apparent maximal response E’ _MAX_ observed as the upper asymptote of the functional response curve is given by Eq. (3).

3 $$E_{MAX}^{\prime } = \frac{{\tau_{A} E_{MAX} }}{{\tau_{A} + 1}}$$

The relationship between EC_50_ and the observed maximal response E'_MAX_ is given by Eq. (4).

4 $$E_{MAX}^{\prime } = E_{MAX} - \frac{{E_{MAX} EC_{50} }}{{K_{A} }}$$

For the derivation of equations, see Supplementary information, Eq. 1 to 5. From Eqs. (2) and (3) it is obvious that parameters E_MAX_, τ_A_ and K_A_ are inter-dependent. The upper asymptote of functional response, E′_MAX_, may be any combination of τ_A_ and E_MAX_, provided their product equals E′_MAX_. The same applies to EC_50_ value that may be any combination of τ_A_ and K_A_ provided that ratio K_A_ to 1 + τ_A_ equals EC_50_. Therefore, for reliable determination of OM parameters of functional response to an agonist, we have proposed a two-step procedure^15^. First, the apparent maximal response E'_MAX_ and half-efficient concentration EC_50_ are determined from a series of concentration–response curves, then the maximal response of the system E_MAX_ and equilibrium dissociation constant K_A_ are determined by fitting Eq. (4) to E'_MAX_ vs. EC_50_ values. Equation (1) is then fitted to the concentration–response curves with fixed E_MAX_ and K_A_ values to determine values of operational efficacy, τ_A_.

### Allosteric modulation

An allosteric modulator is a ligand that binds to a site on the receptor that is spatially distinct from that of endogenous agonists and orthosteric ligands. Both agonist A and allosteric modulator B can bind to the receptor R simultaneously and form a ternary complex ARB (Figs. 1 and 2). The equilibrium dissociation constant K_A_ of an agonist A to the binary complex RB of the allosteric modulator and receptor differs from the equilibrium dissociation constant of agonist binding in the absence of allosteric modulator, K_A_, by a factor of binding cooperativity α (K_A_/α). The law of microscopic reversibility of thermodynamics dictates that the equilibrium dissociation constant of an allosteric modulator K_B_ to the binary complex AR of agonist and receptor differs from K_B_ by the same factor α (K_B_/α). Values of the factor of binding cooperativity α greater than unity denote positive cooperativity, where binding of agonist and allosteric modulator mutually strengthens each other. Values of the factor of binding cooperativity α lower than 1 denote negative cooperativity, where binding of agonist and allosteric modulator mutually reduces the affinity of each other.

The thermodynamically complete description of allosteric modulation of receptor activation is described by the CTC model (Fig. 1)^16^. Although the CTC model is simplified and omits improbable interactions of inactive-receptor complexes with G-proteins, besides modulation of binding affinity (equilibrium dissociation constant K_A_), an allosteric ligand may affect the receptor activation constant (K_ACT_). The CTC model of binding and activation (Fig. 1, left cube) therefore consists of three equilibrium constants and four factors of cooperativity. The allosteric modulator may also affect the affinity of the receptor complex for G-protein, K_G_, and efficacy of G-protein activation, φ^25–27^. Thus, it is obvious that such heuristic models are too complex to estimate any of their parameters.

The practical way to analyse allosteric modulation of pharmacological agonism is a parsimonious operational model where the effects of allosteric modulators on operational efficacy are quantified by the operational factor of cooperativity, β (Fig. 2). In this model the operational efficacy of the ternary complex of agonist, receptor and allosteric modulator, ARB, is β*τ_A_. Values of operational cooperativity β greater than 1 denote positive cooperativity; the functional response to an agonist in the presence of allosteric modulator is greater than in its absence. Values of operational cooperativity β lower than 1 denote negative cooperativity, where the functional response to an agonist in the presence of an allosteric modulator is smaller than in its absence.

The functional response to an agonist in the presence of an allosteric modulator is given by Eq. (5)^17^.

5 $$Response = \frac{{E_{MAX} \tau_{A} \left[ A \right]\left( {K_{B} + \alpha \beta \left[ B \right]} \right)}}{{\left[ A \right]K_{B} + K_{A} K_{B} + \left[ B \right]K_{A} + \alpha \left[ A \right]\left[ B \right] + \tau_{A} \left[ A \right]\left( {K_{B} + \alpha \beta \left[ B \right]} \right)}}$$

where [A] and [B] are the concentrations of an agonist and allosteric modulator, respectively, E_MAX_ is the maximal response of the system, K_A_ and K_B_ are the equilibrium dissociation constants of the agonist-receptor and allosteric modulator-receptor complex, respectively, and τ_A_ is the operational factor of efficacy of an agonist. As can be seen, even Eq. (5) is difficult to fit the functional response data directly. As we have shown previously^15^, all three parameters of OM (Eq. (1)), E_MAX_, K_A_ and τ_A_ are inter-dependent. Therefore, they cannot be reliably determined by fitting of Eq. (1) to functional response data. These parameters are also inter-dependent in Eq. (5). Moreover, this equation is more complex than Eq. (1). Below we analyse the operational model of allosterically-modulated agonism (OMAM) by the same approach used previously for the OM^15^.

From Eq. (5) apparent half-efficient concentration of an agonist, EC'_50_, is given by Eq. (6) and the apparent maximal response induced by an agonist, E'_MAX_, is given by Eq. (7). Alternative expressions of EC′_50_ and E′_MAX_ can be found in Supplementary Information.

6 $$EC_{50}^{\prime } = \frac{{K_{A} \left( {\left[ B \right] + K_{B} } \right)}}{{\alpha \left[ B \right] + \left( {\alpha \beta \left[ B \right] + K_{B} } \right)\tau_{A} + K_{B} }}$$

7 $$E_{MAX}^{\prime } = \frac{{\left( {\alpha \beta \left[ B \right] + K_{B} } \right)\tau_{A} E_{MAX} }}{{\alpha \left[ B \right] + \left( {\alpha \beta \left[ B \right] + K_{B} } \right)\tau_{A} + K_{B} }}$$

From Eq. (6) it is obvious that both factors α and β affect EC′_50_. Thus, α and β are the fourth and fifth inter-dependent parameters with τ_A_, E_MAX_ and K_A_. Equation (7) indicates that the factor of operational cooperativity β affects observed maximal response E′_MAX_. For saturation concentrations of an allosteric modulator B Eq. (7) becomes Eq. (8).

8 $$E_{MAX}^{\prime } = \frac{{\beta \tau_{A} E_{MAX} }}{{\beta \tau_{A} + 1}}$$

The factor of binding cooperativity, α, can be determined from the dependence of the dose ratio of EC_50_ values on the concentration of allosteric modulator. The dose ratio of EC_50_ in the absence of allosteric modulator to EC'_50_ in its presence at the concentration [B] is given by Eq. (8).

9 $$\frac{{EC_{50} }}{{EC_{50}^{\prime } }} = \frac{{\alpha \left[ B \right] + \left( {K_{B} + \alpha \beta \left[ B \right]} \right)\tau_{A} + K_{B} }}{{\left( {\tau_{A} + 1} \right)\left( {K_{B} + \left[ B \right]} \right)}}$$

Values of the ratio greater than 1 where EC'_50_ is lower than EC_50_, denote an increase in potency mediated by positive cooperativity. Ratio values smaller than 1 denote negative cooperativity and a decrease in potency.

In case the allosteric modulator does not affect operational efficacy τ_A_ (β = 1), Eq. (9) simplifies to Eq. (10).

10 $$\frac{{EC_{50} }}{{EC_{50}^{\prime } }} = \frac{{K_{B} + \alpha \left[ B \right]}}{{K_{B} + \left[ B \right]}}$$

**Figure 3:**
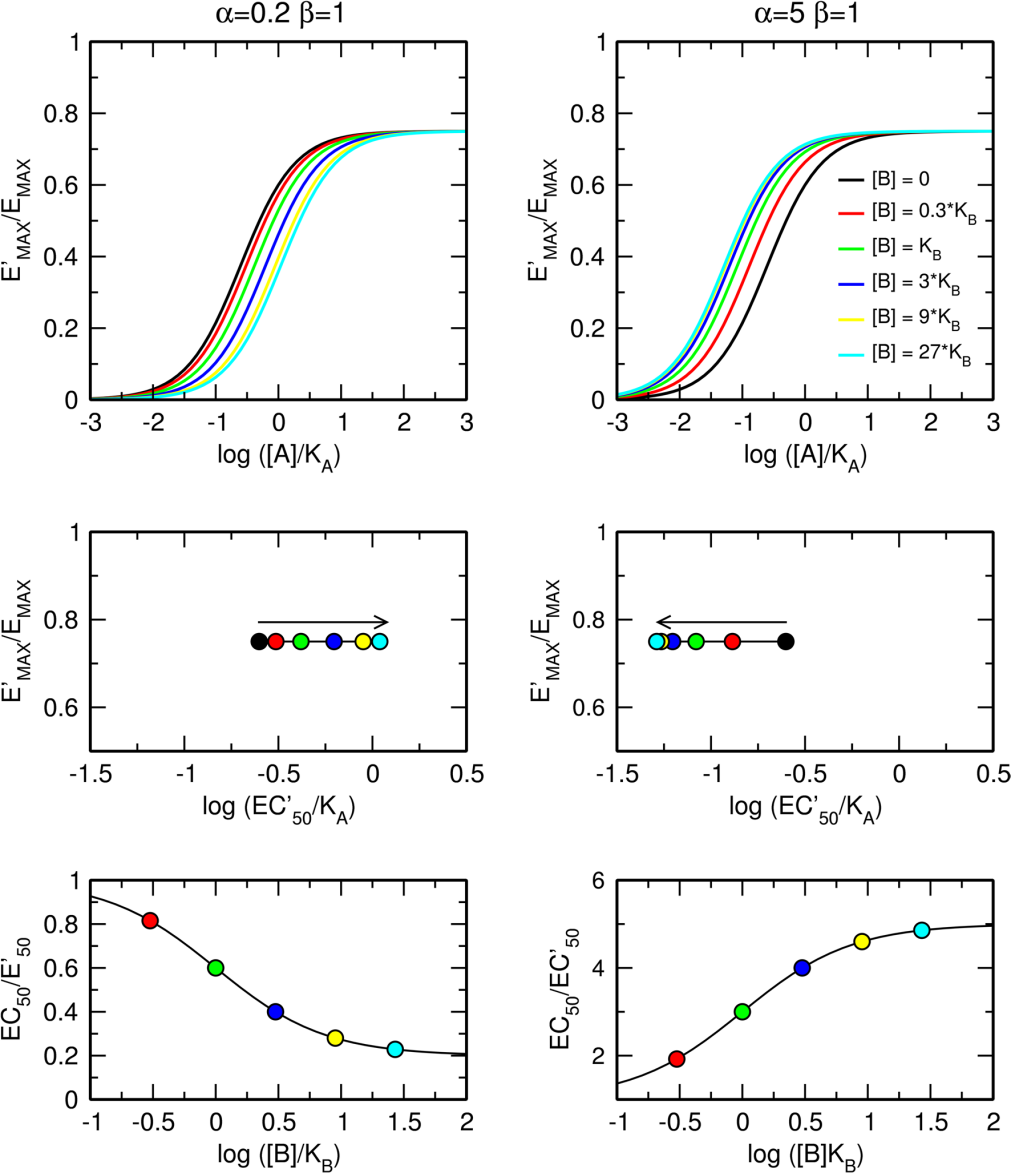
Effects of a pure allosteric modulator on functional response to an orthosteric agonist. Effects of a pure negative (left) and positive (right) allosteric modulator on functional response to orthosteric agonist (top); observed half-efficient concentration EC'_50_ and observed maximal response E'_MAX_ (middle); and dose ratio of half-efficient concentrations (bottom). E_MAX_ = 1, τ_A_ = 3, values of factors of cooperativity α and β are indicated within the plots.

The factor of binding cooperativity α affects only the apparent half-efficient concentration, EC'_50_, of an agonist (Fig. 3). In the case of negative cooperativity (Fig. 3, left), the allosteric modulator concentration-dependently increases the value of EC'_50_ without a change in the apparent maximal response, E'_MAX_. In the case of positive cooperativity (Fig. 3, right), the allosteric modulator decreases EC'_50_ without a change in E'_MAX_. The maximal dose ratio is equal to α as for [B] much greater than K_B_, the right side of Eq. (10) becomes equal to α (Fig. 3, bottom).

In case the allosteric modulator does not affect the equilibrium dissociation constant of an agonist K_A_ (α = 1), Eq. (9) simplifies to Eq. (11).

11 $$\frac{{EC_{50} }}{{EC_{50}^{\prime } }} = \frac{{\tau_{A} \left( {\beta \left[ B \right] + K_{B} } \right) + \left[ B \right] + K_{B} }}{{\tau_{A} \left( {\left[ B \right] + K_{B} } \right) + \left[ B \right] + K_{B} }}$$

**Figure 4:**
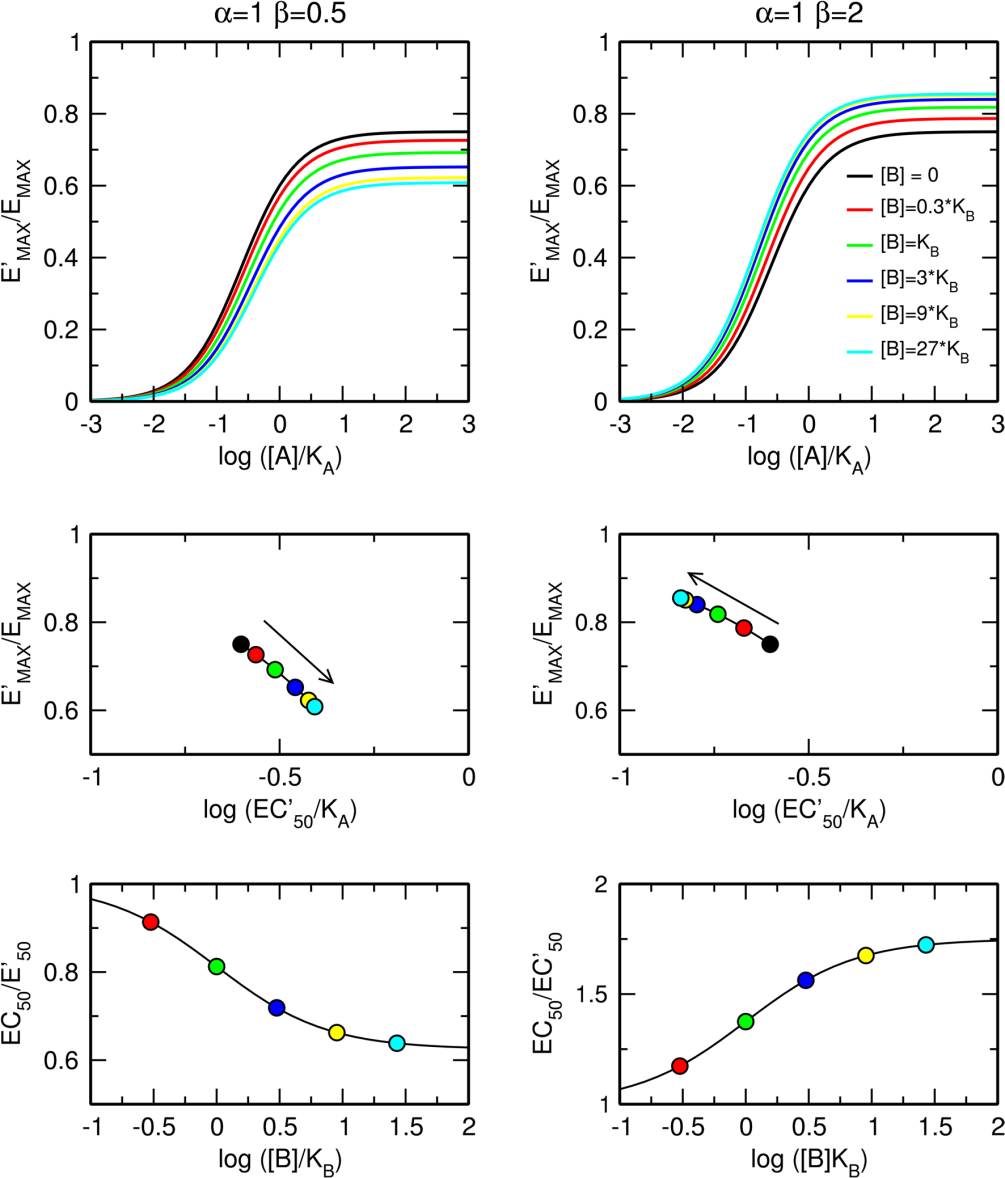
Effects of a silent allosteric modulator on the functional response to an orthosteric agonist. Effects of a pure negative (left) and positive (right) allosteric modulator on the functional response to an orthosteric agonist (top); observed half-efficient concentration EC'_50_ and observed maximal response E'_MAX_ (middle); and dose ratio of half-efficient concentrations (bottom). E_MAX_ = 1, τ_A_ = 3, values of factors of cooperativity α and β are indicated within the plots.

In contrast to the factor of binding cooperativity α, the factor of operational cooperativity β affects both the observed maximal response E'_MAX_ and the observed half-efficient concentration of an agonist EC'_50_ (Fig. 4). In the case of negative operational cooperativity (Fig. 4, left), the allosteric modulator concentration-dependently increases the value of EC'_50_ and decreases the observed maximal response E'_MAX_. In the case of positive operational cooperativity (Fig. 4, right), the allosteric modulator decreases EC'_50_ and increases E'_MAX_. The maximal dose ratio is given by Eq. (12) (Fig. 4, bottom).

12 $$For \left[ B \right] \gg K_{B} ;\, \frac{{EC_{50} }}{{EC_{50}^{\prime } }} = \frac{{\beta \tau_{A} + 1}}{{\tau_{A} + 1}}$$

**Figure 5:**
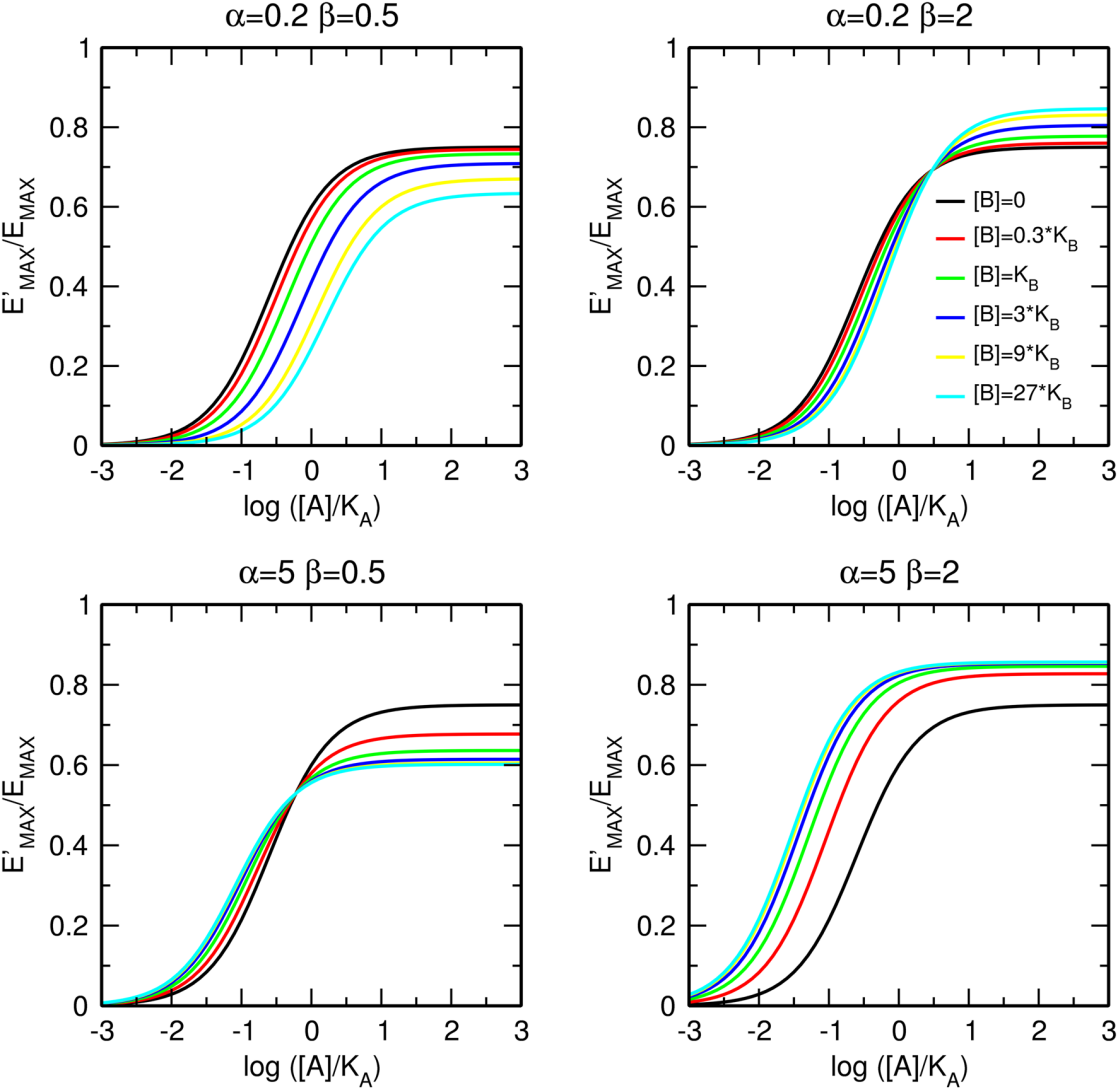
Combined effects of the allosteric modulator on binding affinity and operational efficacy. Effects of positive (top) and negative (bottom) modulation of binding affinity and negative (left) and positive (right) modulation of operational efficacy. E_MAX_ = 1, τ_A_ = 3, values of factors of cooperativity α and β are indicated within the plots. Meta-analysis of concentration response curves is in Supplementary information Figure S1 and S2.

Additional combinations of types of cooperativity of binding, α, and operational efficacy, β, between an orthosteric agonist and allosteric modulator are illustrated in Fig. 5. The meta-analysis of concentration–response curves is in Supplementary information Figure S1 and S2. For the derivation of equations, see Supplementary information, Eqs. 6 to 29.

### Allosteric agonists

The allosteric ligand may possess its own intrinsic activity, i.e., being able to activate the receptor in the absence of an agonist. Such allosteric ligand is termed allosteric agonist. According to the OM, the response to an allosteric agonist is given by Eq. (13).

13 $$Response = \frac{{\left[ B \right]\tau_{B} E_{MAX} }}{{\left[ B \right]\left( {\tau_{B} + 1} \right) + K_{B} }}$$

where [B] is the concentration of an allosteric agonist, E_MAX_ is the maximal response of the system, K_B_ is the equilibrium dissociation constant of the complex of allosteric agonist and receptor and τ_B_ is the operational factor of efficacy of the allosteric modulator. In the presence of an allosteric modulator, the response to an orthosteric agonist is given by Eq. (14).

14 $$Resp = \frac{{E_{MAX} \left( {\tau_{A} \left[ A \right]\left( {K_{B} + \alpha \beta \left[ B \right]} \right) + \tau_{B} \left[ B \right]K_{A} } \right)}}{{\left[ A \right]K_{B} + K_{A} K_{B} + \left[ B \right]K_{A} + \alpha \left[ A \right]\left[ B \right] + \tau_{A} \left[ A \right]\left( {K_{B} + \alpha \beta \left[ B \right]} \right) + \tau_{B} \left[ B \right]K_{A} }}$$

Equation (14) is even more complex than Eq. (5). From Eq. (14) the apparent half-efficient concentration of an agonist, EC'_50_, is given by Eq. (15) and apparent maximal response induced by an agonist, E'_MAX_, is given by Eq. (16). Alternative expressions of EC′_50_ and E′_MAX_ can be found in Supplementary Information, Eq. 38, 41 and 42.

15 $$EC_{50}^{\prime } = \frac{{K_{A} \left( {\left[ B \right] + K_{B} } \right) + \tau_{B} \left[ B \right]K_{B} }}{{\alpha \left[ B \right] + \left( {K_{B} + \alpha \beta \left[ B \right]} \right)\tau_{A} + K_{B} }}$$

16 $$E_{MAX}^{\prime } = \frac{{\left( {K_{B} + \alpha \beta \left[ B \right]} \right)\tau_{A} E_{MAX} }}{{\alpha \left[ B \right] + \left( {K_{B} + \alpha \beta \left[ B \right]} \right)\tau_{A} + K_{B} }}$$

Apparent maximal response E′_MAX_ to an agonist in the presence of allosteric agonist at saturation concentration is independent of operational efficacy of allosteric agonist τ_B_ and thus is given by Eq. (8). The dose ratio of EC_50_ in the absence of allosteric modulator to EC'_50_ in its presence at concentration [B] is given by Eq. (17).

17 $$\frac{{EC_{50} }}{{EC_{50}^{\prime } }} = \frac{{\alpha \beta \left[ B \right]\tau_{A} + \alpha \left[ B \right] + K_{B} \tau_{A} + K_{B} }}{{\left( {\tau_{A} + 1} \right)\left( {\left[ B \right]\tau_{B} + \left[ B \right] + K_{B} } \right)}}$$

To separate individual factors of cooperativity, the dose ratio may be expressed by Eq. (18).

18 $$\frac{{EC_{50} }}{{EC_{50}^{\prime } }} = \frac{{E_{MAX} \left( {\alpha \left[ B \right] + K_{B} } \right)}}{{\left( {\tau_{A} + 1} \right)\left( {E_{MAX} - E_{MAX}^{\prime } } \right)\left( {\left[ B \right]\tau_{B} + \left[ B \right] + K_{B} } \right)}}$$

**Figure 6:**
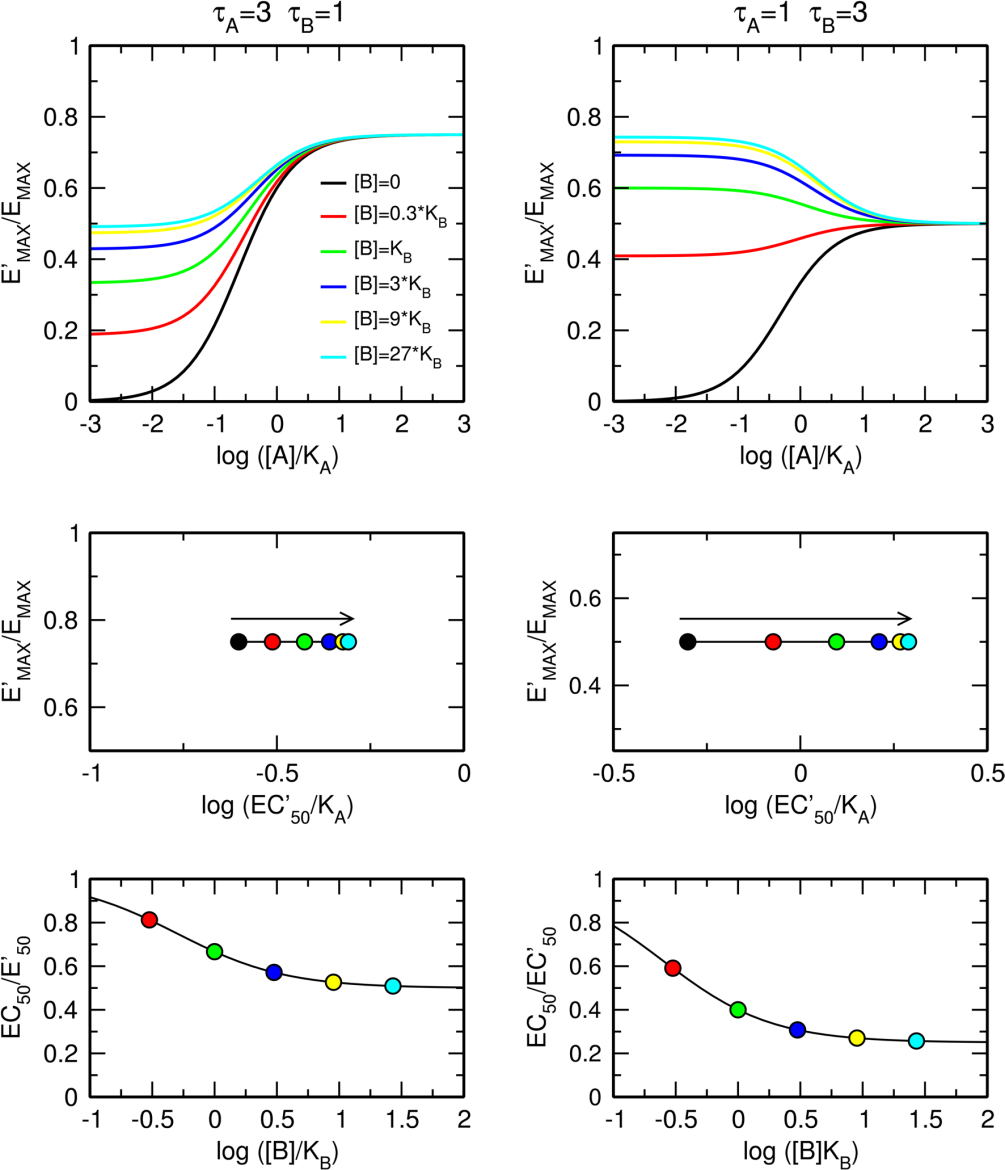
Effects of an allosteric agonist that neither affects affinity (α = 1) nor functional efficacy (β = 1) of an orthosteric agonist. Effects of an allosteric agonist where efficacy is smaller (left) or greater (right) than the efficacy of the orthosteric agonist on the functional response to an orthosteric agonist (top); observed half-efficient concentration EC'_50_ and observed maximal response E'_MAX_ (middle); and dose ratio of half-efficient concentrations (bottom). E_MAX_ = 1, α = 1, β = 1, operational efficacies τ_A_ and τ_B_ of the orthosteric and allosteric agonist, respectively, are indicated within the plots.

The principal difference between an allosteric agonist and allosteric modulator is that the former increases the basal level of functional response on its own. Even if an allosteric agonist exerts neutral binding cooperativity (α = 1) and does not affect the operational efficacy of the orthosteric agonist (β = 1), it increases the half-efficient concentration, EC′_50_, of the orthosteric agonist regardless the ratio of operational efficacies τ_A_ and τ_B_. (Fig. 6). Figure 6 illustrates pure allosteric interaction with τ_B_ lower (left) and greater (right) than τ_A_. As it can be seen in Fig. 6, the observed maximal response, E′_MAX_, is given by the factor of operational efficacy of the orthosteric agonist, τ_A_, according to Eq. (18).

18 $$E_{MAX}^{\prime } = \frac{{\tau_{A} E_{MAX} }}{{\tau_{A} + 1}}$$

The EC′_50_ value depends on the concentration of pure allosteric agonist (α = 1, β = 1) according to Eq. (19).

19 $$EC_{50}^{\prime } = \frac{{K_{A} \left( {\left[ B \right]\tau_{B} + \left[ B \right] + K_{B} } \right)}}{{\left( {\tau_{A} + 1} \right)\left( {\left[ B \right] + K_{B} } \right)}}$$

It is evident from Fig. 6 and Eq. (19) that the factor of operational efficacy of an allosteric modulator, τ_B_, affects the EC′_50_ value of the orthosteric agonist. Thus, τ_B_ is the sixth inter-dependent parameter in addition to α, β, τ_A_, E_MAX_ and K_A_. For a pure allosteric agonist (α = 1, β = 1) Eq. (16) simplifies to Eq. (20).

20 $$\frac{{EC_{50} }}{{EC_{50}^{\prime } }} = \frac{{\left[ B \right] + K_{B} }}{{\left[ B \right]\tau_{B} + \left[ B \right] + K_{B} }}$$

For a pure allosteric agonist the maximal dose ratio is given by Eq. (21) (Fig. 6, bottom).

21 $$For\left[ B \right] \gg K_{B}; \frac{{EC_{50} }}{{EC_{50}^{\prime } }} = \frac{1}{{\tau_{B} + 1}}$$

In case an allosteric agonist does not affect the operational efficacy τ_A_ (β = 1) but allosterically modulates the affinity of the orthosteric agonist (α ≠ 1), Eq. (16) simplifies to Eq. (22).

22 $$\frac{{EC_{50} }}{{EC_{50}^{\prime } }} = \frac{{\alpha \left[ B \right] + K_{B} }}{{\left[ B \right]\tau_{B} + \left[ B \right] + K_{B} }}$$

**Figure 7:**
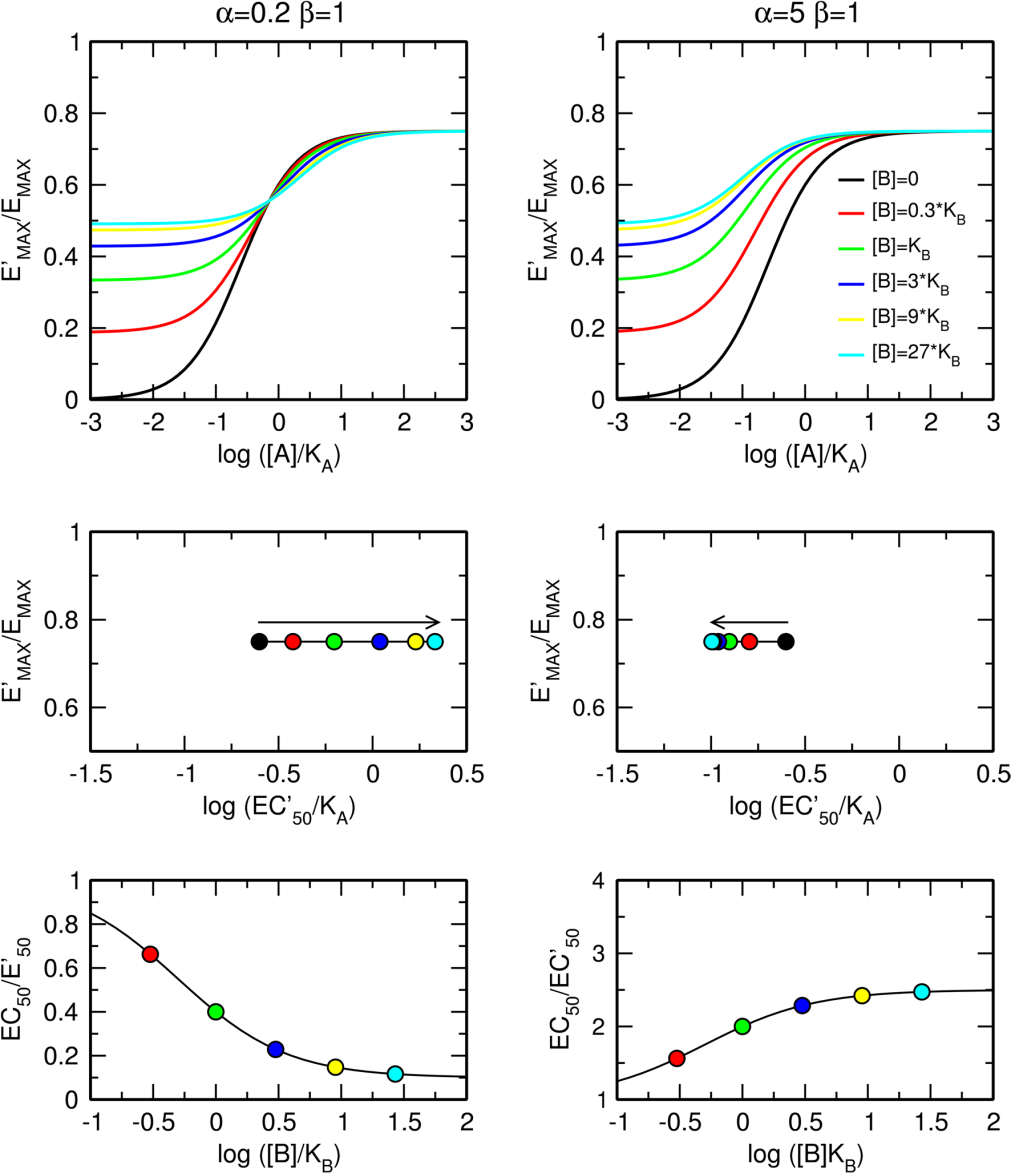
Effects of an allosteric agonist that does not affect efficacy (β = 1) of an orthosteric agonist in producing a functional response. Effects of a pure negative (left) or positive (right) allosteric modulator on the functional response to an orthosteric agonist (top); observed half-efficient concentration EC'_50_ and observed maximal response E'_MAX_ (middle); and dose ratio of half-efficient concentrations (bottom). E_MAX_ = 1, τ_A_ = 3, τ_B_ = 1, values of factors of cooperativity α and β are indicated within the plots.

The factor of binding cooperativity α affects only the apparent half-efficient concentration, EC'_50_, of an orthosteric agonist (Fig. 7). In the case of negative cooperativity (Fig. 7, left), an allosteric agonist concentration-dependently increases the value of EC'_50_ without a change in apparent maximal response E'_MAX_. In the case of positive cooperativity (Fig. 7, right), an allosteric agonist decreases EC'_50_ without a change in E'_MAX_. For an allosteric agonist with neutral operational cooperativity (β = 1), the maximal dose ratio is given by Eq. (23) (Fig. 7, bottom).

23 $$For \left[ B \right] \gg K_{B} ; \frac{{EC_{50} }}{{EC_{50}^{\prime } }} = \frac{\alpha }{{\tau_{B} + 1}}$$

In case allosteric agonist does not affect equilibrium dissociation constant of an orthosteric agonist K_A_ (α = 1) but allosterically modulates the operational efficacy of the orthosteric agonist (β ≠ 1) Eq. (16) simplifies to Eq. (24).

24 $$\frac{{EC_{50} }}{{EC_{50}^{\prime } }} = \frac{{\left[ B \right] + \beta \left[ B \right]\tau_{A} + K_{B} \tau_{A} + K_{B} }}{{\left( {\tau_{A} + 1} \right)\left( {\left[ B \right] + \left[ B \right]\tau_{B} + K_{B} } \right)}}$$

**Figure 8:**
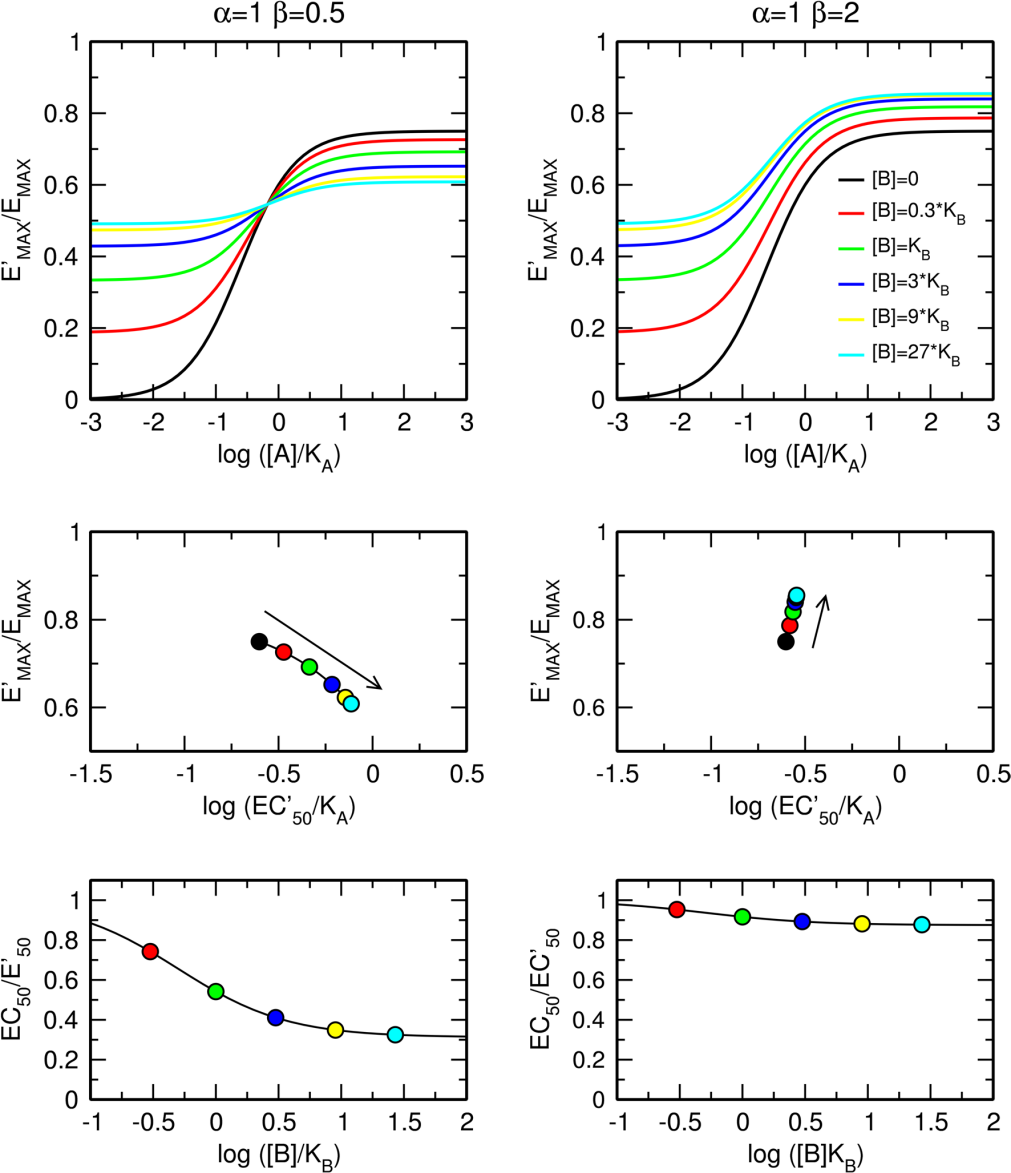
Effects of an allosteric agonist that does not affect the affinity (α = 1) of an orthosteric agonist in producing a functional response. Effects of a pure negative (left) or positive (right) allosteric modulator on the functional response to an orthosteric agonist (top); observed half-efficient concentration EC'_50_ and observed maximal response E'_MAX_ (middle); and dose ratio of half-efficient concentrations (bottom). E_MAX_ = 1, τ_A_ = 3, τ_B_ = 1, values of factors of cooperativity α and β are indicated within the plots.

In contrast to the factor of binding cooperativity, α, the factor of operational cooperativity, β, affects both the observed maximal response E'_MAX_ and observed half-efficient concentration of an agonist EC'_50_ (Fig. 8). In the case of negative operational cooperativity (Fig. 8, left), the allosteric agonist concentration-dependently decreases the observed maximal response E'_MAX_ and increases the value of EC'_50_. In the case of positive operational cooperativity (Fig. 8, right), the allosteric agonist increases E'_MAX_ but, paradoxically, may increase EC'_50_ as shown in Fig. 8, right. This increase in EC'_50_ value happens when a decrease in the EC'_50_ value due to positive operational cooperativity β is smaller than an increase in the EC'_50_ value due to operational activity of allosteric agonist τ_B_. Maximal dose ratio is given by Eq. (25) (Fig. 8, bottom).

25 $$For \left[ B \right] \gg K_{B} ; \frac{{EC_{50} }}{{EC_{50}^{\prime } }} = \frac{{\beta \tau_{A} + 1}}{{\left( {\tau_{A} + 1} \right)\left( {\tau_{B} + 1} \right)}}$$

**Figure 9:**
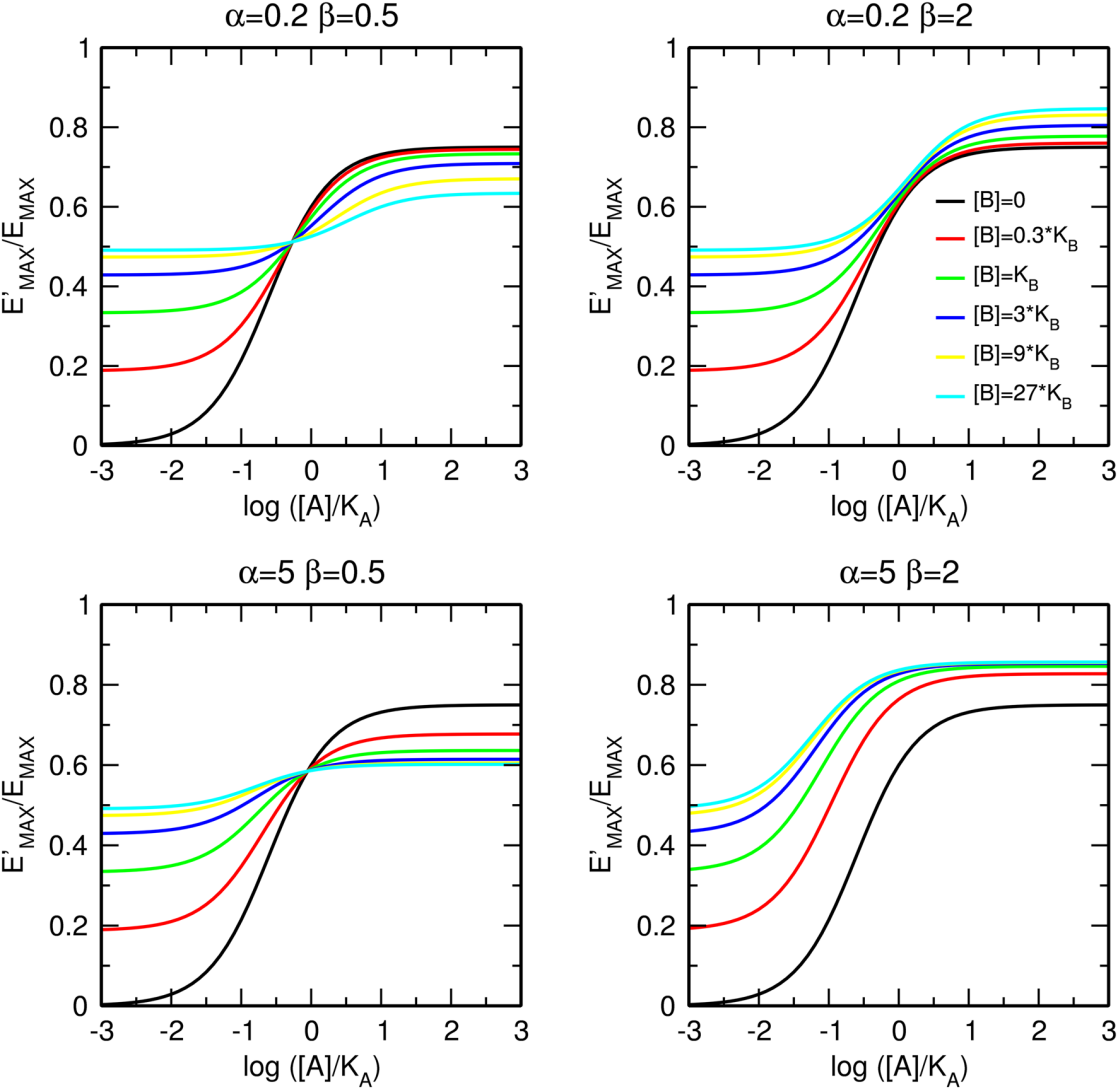
Combined effects of allosteric agonist on binding affinity and operational efficacy. Effects of positive (top) and negative (bottom) modulation of binding affinity and negative (left) and positive (right) modulation of operational efficacy. E_MAX_ = 1, τ_A_ = 3, τ_B_ = 1, values of factors of cooperativity α and β are indicated within the plots. Meta-analysis of concentration–response curves is in Supplementary information Figs. S3 and S4.

Additional combinations of types of cooperativity of binding α and operational efficacy β between orthosteric and allosteric agonists are illustrated in Fig. 9. The meta-analysis of concentration–response curves is in Supplementary information Figure S3 and S4. For the derivation of equations, see Supplementary information, Eqs. 30 to 56.

## Application of models to experimental data

**Figure 10:**
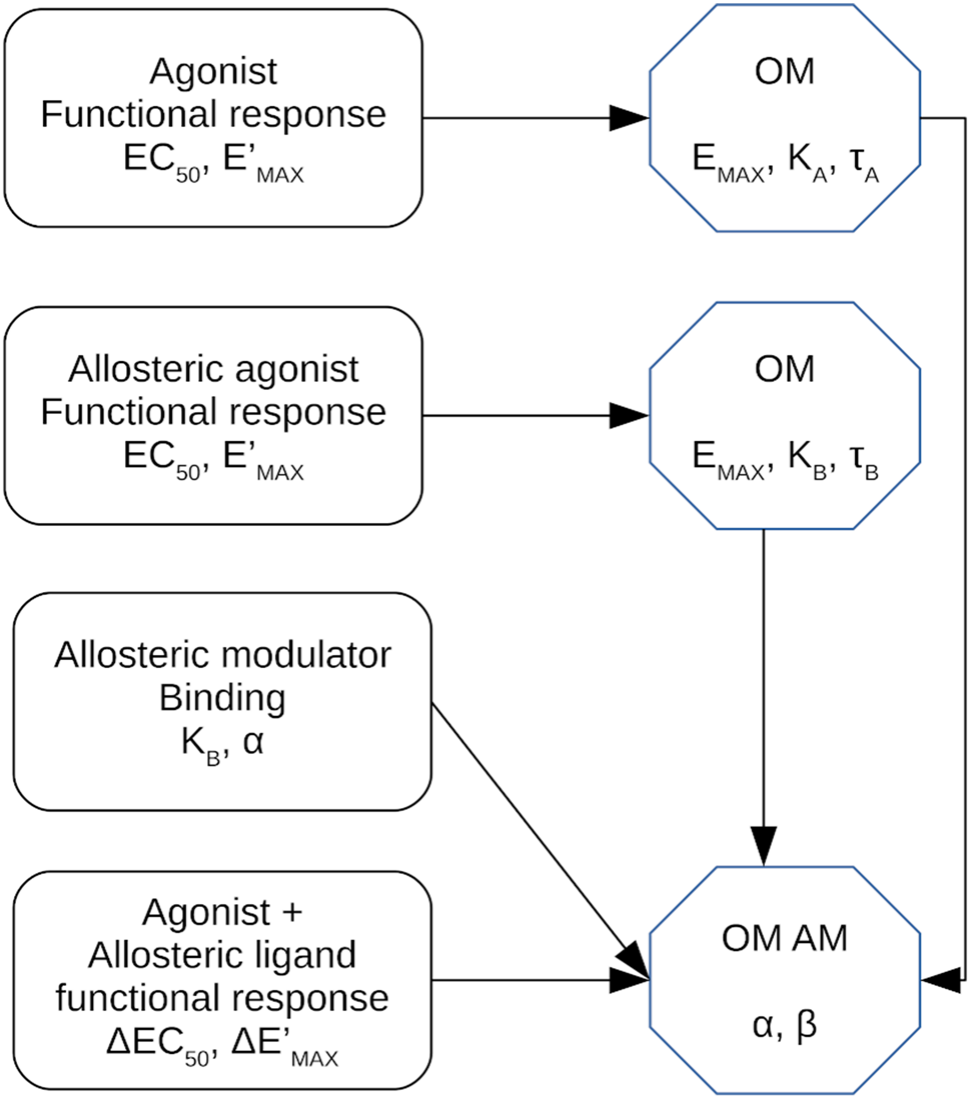
Analysis workflow of allosteric modulation of pharmacological agonism. The following parameters should be determined in respective experiments before fitting Eq. (5) that describes the operational model of allosterically-modulated agonism (OMAM) to the functional response data. The maximal response of the system E_MAX_ and agonist equilibrium dissociation constant K_A_ and operational efficacy τ_A_ are determined in functional experiments. The equilibrium dissociation constant of allosteric modulator K_B_ and factor of binding cooperativity α are obtained from binding experiments. In the case of an allosteric agonist, its equilibrium dissociation constant K_B_ and operational efficacy τ_B_ are determined in functional experiments.

In this section, we demonstrate the application of OMAM equations described above to experimental data. The allosteric modulation of M_1_ muscarinic receptor by benzyl quinolone carboxylic acid (BQCA) and 3-[1-[1-[(2-methylphenyl)methyl]piperidin-4-yl]piperidin-4-yl]-1H-benzimidazol-2-one (TBPB) was investigated. Agonist carbachol and super-agonist iperoxo were used to stimulate the level of inositol phosphates (IP_X_). Individual parameters of OMAM with high confidence were obtained following the flowchart in Fig. 10. First, binding parameters (K_A_, K_B_ and α) and parameters of functional response to sole orthosteric agonists (K_A_, τ_A_) as well as possible allosteric agonists (K_B_ and τ_B_) were determined. Then these parameters were used in the determination of cooperativity factors α and β according to OMAM.

### Binding parameters

**Figure 11:**
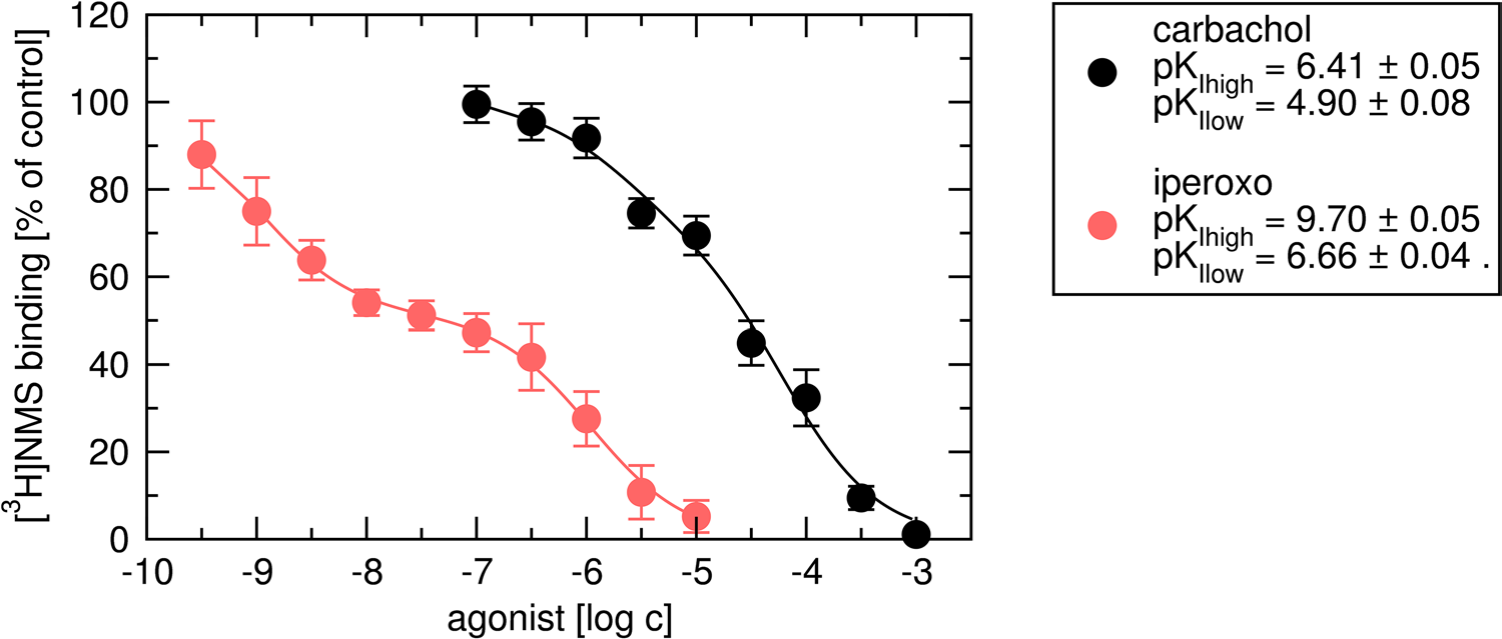
Determination of agonist inhibitory constants. Binding of [^*3*^H]NMS in the presence of carbachol (black) or iperoxo (red) is expressed as per cent of binding in the absence of an agonist. Concentrations of agonists are expressed as logarithms. Data are means ± SD from a representative experiment. Calculated negative logarithms of inhibitory constants ± SD are indicated in the legend. Parameters were obtained by fitting Eq. (27) to the data and calculation of K_I_ values from IC_50_ values according to Eq. (28). Parameters are summarised in Supplementary information Table S1.

**Figure 12:**
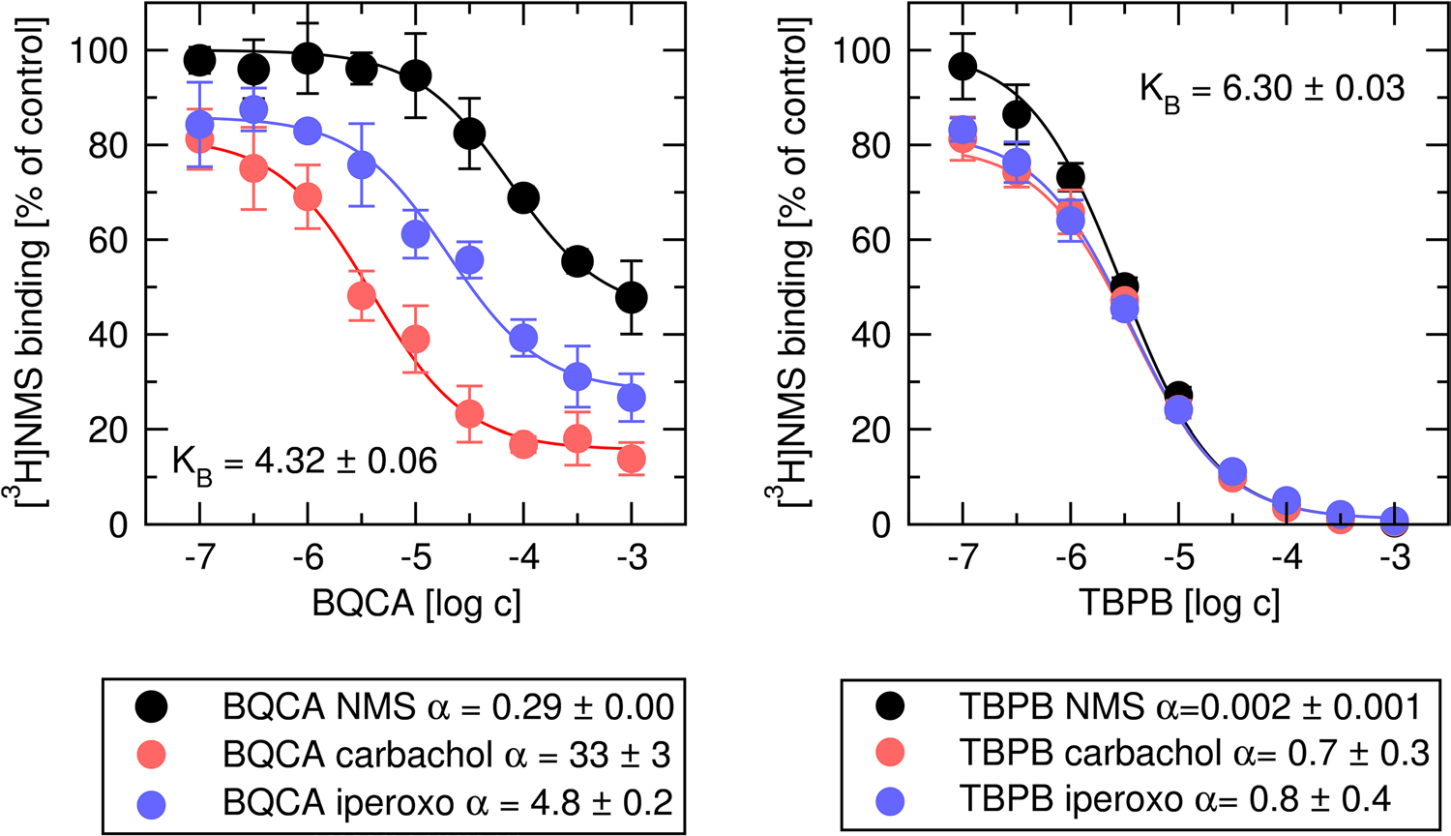
Determination of binding parameters of allosteric modulators. Binding of [^3^H]NMS in the presence of BQCA (left) or TBPB (right) alone (black) or in combination with carbachol (red) or iperoxo (blue) is expressed as per cent of binding in the absence of an allosteric modulator and agonist. Concentrations of the allosteric modulator are expressed as logarithms. Data are means ± SD from a representative experiment. Calculated negative logarithms of equilibrium dissociation constants K_A_ ± SD and are indicated in the plots. Calculated factors of binding cooperativity α ± SD are indicated in the legend. Parameters were obtained by fitting Eqs. (27) and (28) as appropriate. Parameters are summarised in Supplementary information Table S2.

The binding parameters were determined in competition experiments with the radiolabelled orthosteric antagonist N-methylscopolamine ([^3^H]NMS). Both agonists, carbachol and iperoxo, displayed binding to two populations of binding sites (Fig. 11). Their high-affinity binding of 390 nM and 200 pM, respectively, can be taken as candidate values for equilibrium dissociation constants K_A_ in OM. It should be noted that not always high-affinity binding site corresponds to the conformation of the receptor that initiates the signalling^15^. Allosteric modulator BQCA caused incomplete inhibition of [^3^H]NMS indicating the allosteric mode of interaction (Fig. 12, left). BQCA decreased affinity of [^3^H]NMS more than threefold. BQCA increased affinity of carbachol 33-times and iperoxo 4.8-times. BQCA apparent K_B_ was about 50 μM. In contrast to BQCA, putative allosteric modulator TBPB completely inhibited [^3^H]NMS binding making it impossible to tell whether the interaction between [^3^H]NMS and TBPB is allosteric or competitive (Fig. 12, right). Fitting Eq. (29) resulted in the estimation of 500-fold decrease in [^3^H]NMS affinity with high uncertainty. The apparent K_B_ of TBPB was 500 nM. According to fitting Eq. (30) cooperativity between TBPB and carbachol and iperoxo was slightly negative with a high degree of uncertainty (Supplementary information Table S2).

### Parameters of OM

**Figure 13:**
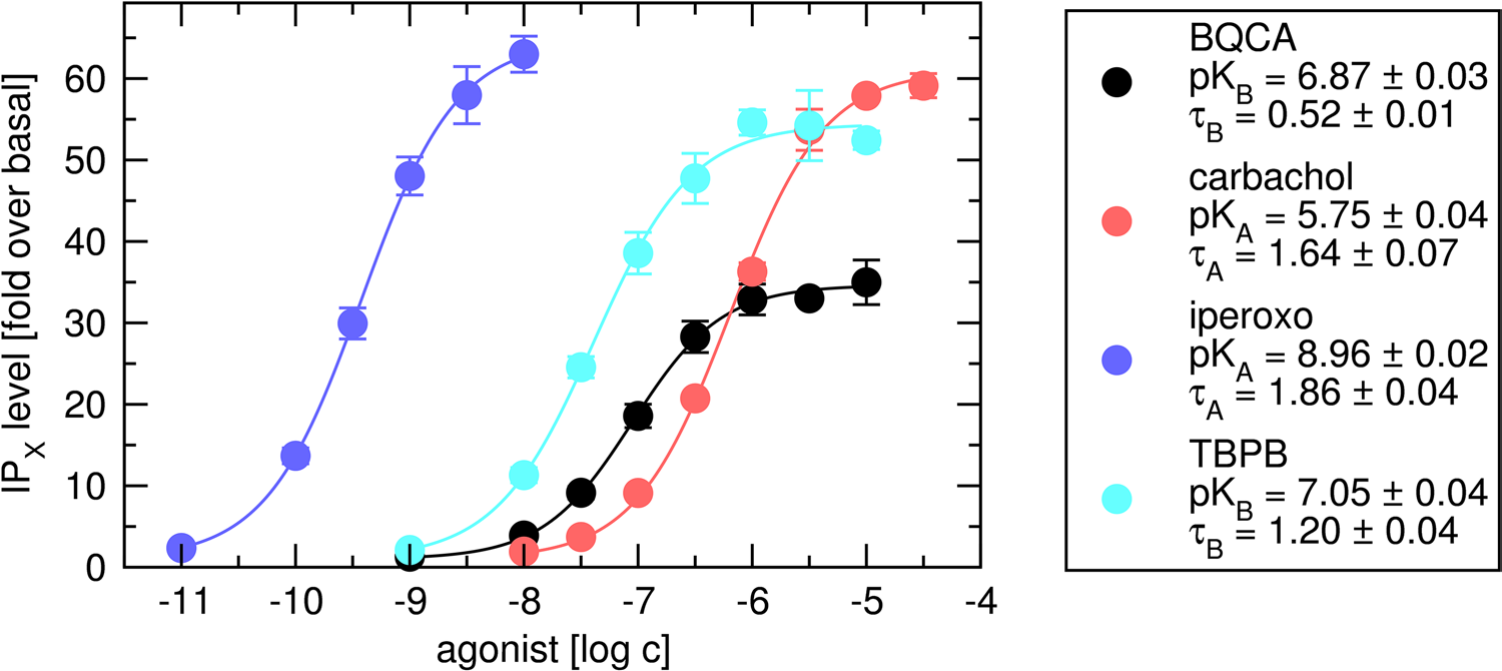
Determination of parameters of functional response to agonists and allosteric modulators. Level of inositol phosphates (IP_X_) stimulated by agonist carbachol (red) or iperoxo (blue) or allosteric modulator BQCA (black) or TBPB (cyan) is expressed as fold-over basal level. Concentrations of agonists and allosteric modulator are expressed as logarithms. Data are means ± SD from a representative experiment. Calculated factors of operational efficacy τ ± SD and the negative logarithm of equilibrium dissociation constants K_A_ ± SD are indicated in the legend. Parameters were obtained by fitting Eq. (1) with E_MAX_ fixed to the predetermined value (E_MAX_ = 98) to the data. Parameters are summarised in Supplementary information Table S3.

The parameters of functional response to sole orthosteric agonists carbachol or iperoxo (K_A_, τ_A_) and allosteric ligands BQCA and TBPB (K_B_ and τ_B_) were determined from changes in intracellular IP_X_ levels in the presence of these ligands (Fig. 13). The basal level of IP_X_ in the absence of agonist was about 0.88% of incorporated radioactivity. Both agonists produced immense response increasing the IP_X_ level more than 50-times. Both allosteric ligands increased level of IP_X_. The E′_MAX_ of response to TBPB was close to E′_MAX_ of full agonist carbachol. First, the logistic Eq. (31) was fitted to the data. The slope of response curves was equal to unity in all cases (Supplementary information Table S3). Maximal system response E_MAX_ determined by the procedure described earlier using a batch of agonists with a full spectrum of efficacies^15^ was 98-fold over basal corresponding to 85% of incorporated radioactivity. The values of τ_A_ were calculated from E_MAX_ and E′_MAX_ values according to Eq. (3). Values of K_A_ were calculated from EC_50_ and τ_A_ values according to Eq. (2). Equation (1) with fixed E_MAX_ was fitted to the data. Initial estimates of K_A_ and τ_A_ were set to calculated values. Resulting K_A_ values of agonists are similar to K_I_ values of high-affinity binding. In contrast, for allosteric ligands, K_B_ values determined in functional experiments (Fig. 13) were more than 3-times higher than K_B_ values determined in binding experiments (Fig. 12). Global fitting of Eq. (1) to all 4 response curves with E_MAX_ as shared parameter resulted in extremely high SD values (Supplementary information Table S3B).

### Cooperativity factors

**Figure 14:**
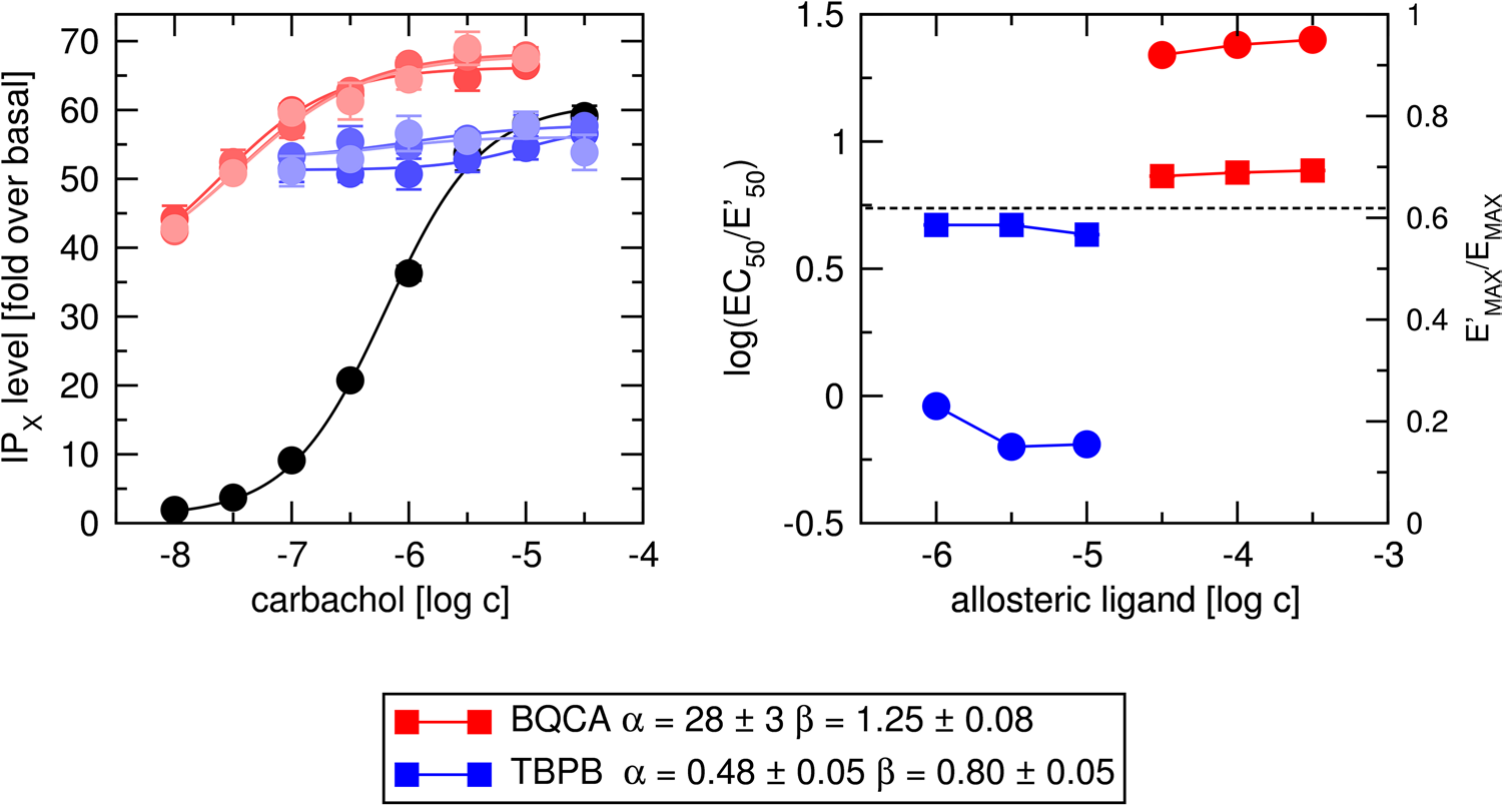
Determination of parameters of allosteric modulation of functional response to carbachol. Left, level of inositol phosphates (IP_X_) stimulated by agonist carbachol alone (black) or in combination with 30, 100 and 300 μM BQCA (shades of red) or 1, 3 and 10 μM TBPB (shades of blue) is expressed as fold-over basal level. Concentrations of carbachol are expressed as logarithms. Right, ratios of half-efficient concentrations EC_50_/ EC′_50_ expressed as logarithms (circles, left y-axis) and observed maximal responses E′_MAX_ /E_MAX_ (squares, right y-axis) are plotted against used concentrations of BQCA (red) and TBPB (blue), respectively. The dashed line indicates E′_MAX_ /E_MAX_ of carbachol. Data are means ± SD from a representative experiment. Calculated factors of binding cooperativity α ± SD and operational cooperativity β ± SD are indicated in the legend. Parameters are summarised in Supplementary information Table S4.

**Figure 15:**
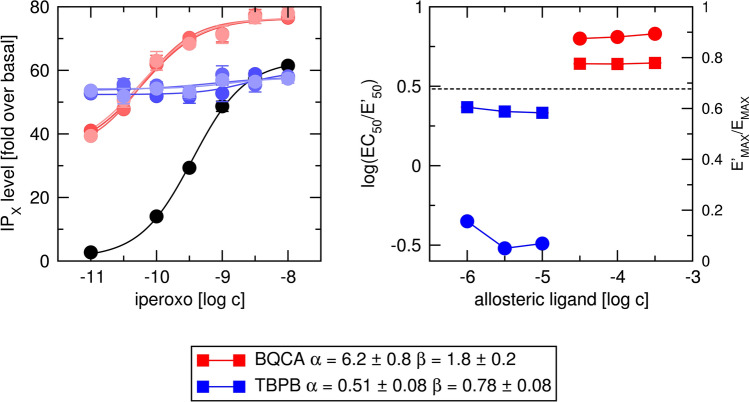
Determination of parameters of allosteric modulation of functional response to iperoxo. Left, level of inositol phosphates (IP_X_) stimulated by agonist iperoxo alone (black) or in combination with 30, 100 and 300 μM BQCA (shades of red) or 1, 3 and 10 μM TBPB (shades of blue) is expressed as fold-over basal level. Concentrations of carbachol are expressed as logarithms. Right, ratios of half-efficient concentrations EC_50_/ EC′_50_ expressed as logarithms (circles, left y-axis) and observed maximal responses E′_MAX_ /E_MAX_ (squares, right y-axis) are plotted against used concentrations of BQCA (red) and TBPB (blue), respectively. The dashed line indicates E′_MAX_ /E_MAX_ of iperoxo. Data are means ± SD from a representative experiment. Calculated factors of binding cooperativity α ± SD and operational cooperativity β ± SD are indicated in the legend. Parameters are summarised in Supplementary information Table S5.

To determine factors of binding cooperativity α and operational cooperativity β between tested agonists and allosteric ligands, functional response to agonist was carried out in the absence or presence of allosteric ligand at the fixed concentration (Figs. 14 and 15, left). Logistic Eq. (31) was fitted to individual curves and EC_50_/ EC′_50_ and E′_MAX_ /E_MAX_ ratios were plotted (Figs. 14 and 15, right; Supplementary information Tables S4 and S5). BQCA decreased EC′_50_ values and increased E′_MAX_ values of both carbachol (Fig. 14, red) and iperoxo (Fig. 15, red) indicating positive binding cooperativity (α > 1) as well as operational cooperativity (β > 1). BQCA in concentrations 30, 100 and 300 μM had the same effect on both EC′_50_ and E′_MAX_ values indicating saturation of its effect that is the sign of allosteric interaction. TBPB decreased E′_MAX_ values of both carbachol (Fig. 14, blue) and iperoxo (Fig. 15, blue) indicating negative operational cooperativity (β < 1). TBPB increased EC′_50_ values of carbachol and iperoxo. The increase is in part due to TBPB operational efficacy (τ_B_ > 0). Negative binding cooperativity (α < 1) may also contribute to the observed increase in EC′_50_. The effects of TBPB on E′_MAX_ and EC′_50_ were the same at 3 and 10 μM concentration indicating saturation of its effect that is the sign of allosteric interaction.

From E′_MAX_ /E_MAX_ ratios and τ_A_ value (predetermined in Fig. 13) factors of operational cooperativity β were calculated according to Eq. (8). Subsequently, the factor of binding cooperativity α was calculated according to Eq. (15). Calculated cooperativity factors α and β were used as initial guesses in fitting Eq. (13) with E_MAX_, K_A_, K_B_, τ_A_ and τ_B_ fixed to values predetermined in functional experiments (Fig. 13). Global fitting of Eq. (13) to experimental data resulted in small SDs of estimated parameters indicating reliable results. In contrast, global fitting of Eq. (13) to experimental data without fixed K_A_, K_B_, τ_A_ and τ_B_ was impracticable ending with infinite SDs. In the case of BQCA, the factors of binding cooperativity α were the same in binding experiments (Fig. 12) as in functional experiments (Figs. 14 and 15). In the case of TBPB, however, observed negative binding cooperativity was about twice stronger in functional experiments (Figs. 14 and 15) than in binding experiments (Fig. 12).

## Discussion

Proper determination of agonist efficacy is a cornerstone in the assessment of possible agonist selectivity and signalling bias. Apparent agonist efficacy is dependent on the system in which it is determined. The operational model of agonism (OM)^13^ can reliably rank agonist efficacies at any receptor effector system^14^. However, the inherent glitch in OM is that the objective parameters (agonist equilibrium dissociation constant K_A_, its operational efficacy τ_A_ and maximal possible response of the system E_MAX_) describing it are inter-dependent^15^. To circumvent this pitfall, we proposed a two-step procedure of fitting of OM to experimental data. First, E_MAX_ and K_A_ are determined by fitting Eq. (4) to the observed E'_MAX_ and EC_50_ values. Then Eq. (1) is fitted to the concentration–response curves with E_MAX_ and K_A_ fixed to predetermined values. This two-step procedure yields robust fits.

Allosteric modulators are intensively studied for their selectivity and preservation of space–time pattern of signalization they modulate^8–10^. They bind to a receptor concurrently with an orthosteric agonist and change the equilibrium dissociation constant K_A_ of the agonist by a factor of binding cooperativity α (Figs. 1 and 2). Values of binding cooperativity α greater than unity denote positive cooperativity; an increase in binding affinity that is reflected in a decrease in EC'_50_ values (Fig. 3, right). Values of α lower than one denote negative cooperativity; a decrease in binding affinity that is manifested as an increase in EC'_50_ values (Fig. 3, left).

Besides modulation of ligand binding affinity, an allosteric ligand can also affect receptor activation, the affinity of the receptor for G-proteins and efficacy of G-protein activation (Fig. 1)^16,25,26^. Such a multitude of possibilities makes it inconceivable to estimate any of the parameters of heuristic models due to their complexity. In the parsimonious operational model of allosterically-modulated agonism (OMAM) the operational factor of cooperativity β quantifies the overall effect of an allosteric modulator on the operational efficacy of an orthosteric agonist τ_A_ (Fig. 2)^17^. Values of operational cooperativity β greater than one denote positive cooperativity, leading to an increase in the observed maximal response E'_MAX_ (Fig. 4, right). Values of β lower than one denote negative cooperativity, leading to a decrease in E'_MAX_ values (Fig. 4, left).

From Eqs. (6) and (7) it is obvious that both factors α and β are inter-dependent with τ_A_, E_MAX_ and K_A_. Following the logic of the two-step procedure of fitting OM to experimental data described above, the factor of binding cooperativity α should be determined from dose ratios according to Eq. (8), alongside with determination of parameters τ_A_, K_A_ and E_MAX_ before fitting Eq. (5) to experimental data to yield reliable results (Fig. 10).

Some allosteric ligands (termed allosteric agonists) possess own intrinsic activity and activate the receptor in the absence of an agonist^18–24^. The response to an orthosteric agonist in the presence of an allosteric modulator is given by Eq. (14).

In case of allosteric agonists, the observed half-efficient concentration of an orthosteric agonist EC'_50_ is affected not only by factors of cooperativity α and β but also by the operational efficacy of an allosteric agonist τ_B_ (Fig. 6). Thus, the operational efficacy of allosteric agonist τ_B_ becomes the sixth inter-dependent parameter with parameters α, β, τ_A_, K_A_ and E_MAX_. Moreover, as a factor of operational efficacy of the orthosteric agonist τ_A_ is inter-dependent with agonist K_A_, a factor of operational efficacy of the allosteric agonist τ_B_ is inter-dependent with its K_B_. To yield reliable results, operational efficacies τ_A_, τ_B_, equilibrium dissociation constants K_A_ and K_B_, the factor of binding cooperativity α and the maximal response of the system E_MAX_ should be determined before fitting Eq. (14) to experimental data (Fig. 10). Similar to the case of an orthosteric agonist, parameters of an allosteric agonist can be determined by fitting Eq. (4) to the observed E'_MAX_ and EC_50_ values of functional response to the allosteric agonist. The factor of operational cooperativity β can be calculated (no regression necessary) according to Eq. (8). Subsequently, the factor of binding cooperativity α can be calculated (no regression necessary) according to Eqs. (15) or (17).

Equations (5) and (14) describing the OMAM are markedly more complex than Eq. (1) describing the OM. While OM has 3 inter-dependent parameters, τ_A_, K_A_ and E_MAX_, the OMAM has two additional inter-dependent parameters, α and β. In the case of an allosteric agonist, a sixth inter-dependent parameter, the operational efficacy of allosteric agonist τ_B_, comes into play. Therefore, it is necessary to experimentally determine as many parameters as possible before fitting Eqs. (5) or (14) to the data (Fig. 10). Values of K_A_ and τ_A_ of an orthosteric agonist and values of K_B_ and τ_B_ of an allosteric agonist should be determined in functional experiments as described above. Values of K_B_ and α of an allosteric ligand can be determined in binding experiments^28^. However, it should be noted that both values of binding cooperativity α between an orthosteric agonist and an allosteric modulator and equilibrium dissociation constant of an allosteric modulator K_B_ differ for the low-affinity binding site (inactive receptor) and high-affinity binding site (active receptor)^29^. Thus, values of binding parameters of allosteric modulators measured indirectly, e.g. using a radiolabelled antagonist as a tracer^30^, need not be suitable for the fitting of the OMAM. In case values of K_B_ and α cannot be measured directly in the binding experiment, they can be inferred from dose ratios of functional response to an agonist in the presence of an allosteric modulator as described above.

In practice, the analysis of functional responses may be further complicated by response curves not following rectangular hyperbola (n_H_ ≠ 1). Flat curves may indicate negative cooperativity between two sites or non-equilibrium conditions^31^. Steep curves may indicate positive cooperativity between two sites or assay clipping. Such situations deserve further analysis. However, as n_H_ of logistic Eq. (31) does not affect inflexion point (EC′_50_) or upper asymptote (E′_MAX_) derived equation describing relations of EC′_50_ and E′_MAX_ are valid also for flat or steep response curves. This represents another advantage over the direct fitting of Eqs. (5) and (14) as the introduction of n_H_ brings an additional degree of freedom to them.

As a case study, we present the application of derived equations of OMAM on allosteric modulation of M_1_ receptors. We followed the workflow outlined in Fig. 10 to avoid fitting equations with inter-dependent parameters. Instead, we analysed the effects of ligands on apparent half-efficient concentration EC′_50_ and maximal response E′_MAX_. First, binding parameters (K_I_, K_A_, K_B_ and α) were determined in binding experiments (Figs. 11 and 12). Both agonists displayed two binding sites. The high to low-affinity ratio was greater for iperoxo than for carbachol indicating iperoxo has greater efficacy than carbachol^32^.

Parameters of functional response to sole orthosteric agonists (K_A_, τ_A_) and allosteric agonists (K_B_ and τ_B_) were determined in functional experiments (Fig. 13). To determine values of K_A_ and τ_A_ or K_B_ and τ_B_, logistic Eq. (31) was fitted to the data and the system maximal response E_MAX_ was determined by the procedure described earlier^15^. Then Eqs. (1) or (13) with E_MAX_ fixed to predetermined value was fitted to the response curves. The functional experiments confirmed that iperoxo has greater efficacy than carbachol. Obtained K_A_ values corresponded to high-affinity K_I_s indicating that observed high-affinity sites correspond to receptor conformation initiating the signalling. In contrast, K_B_ values determined in functional experiments (Fig. 13) were higher than K_B_ values determined in binding experiments (Fig. 12) indicating that K_B_ determined in the binding experiments is not K_B_ of the receptor in the conformation that initiates the signalling. Rather it is an inactive conformation induced by the antagonists [^3^H]NMS used as a tracer.

Subsequently, functional response to agonist in the presence of allosteric agonists was measured (Figs. 14 and 15). First logistic Eq. (31) was fitted to the experimental data. The factor of operational cooperativity β was calculated (no regression necessary) from E′_MAX_ /E_MAX_ ratio according to Eq. (8). Then factor of binding cooperativity α was calculated (again no regression necessary) from EC_50_/ EC′_50_ ratio according to Eq. (15). Finally, Eq. (14) with K_A_, K_B_, τ_A_ and τ_B_ fixed to values predetermined in functional experiments (Fig. 13) was fitted to the concentration–response curves (global fit) to determine confidence intervals of cooperativity factors α and β. In the case of BQCA, the factors of binding cooperativity α determined in binding experiments (Fig. 12) were the same as those determined in functional experiments (Figs. 14 and 15). In the case of TBPB, they differed. It should be noted that the estimates of TBPB α values in binding experiments were associated with high SDs as result of complete inhibition of [^3^H]NMS binding by TBPB making estimation of the binding cooperativity between [^3^H]NMS and TBPB unreliable. Low SDs obtained by the presented procedure indicate that estimates of α and β are reliable. In contrast, the fitting Eq. (1) (OM) with three inter-dependent parameters is problematic (Supplementary information Table S3B)^15^. OMAM Eq. (5) possesses five and Eq. (14) possesses six inter-dependent parameters making their direct fitting to the experimental data impossible.

## Conclusions

The described workflow analysis of functional response represents a robust way of fitting the operational model of allosterically-modulated agonism (OMAM) to experimental data. We believe that the workflow and derived equations describing relations among functional response to agonists and parameters of OMAM will be helpful to many for proper analysis of experimental data of allosteric modulation of receptors.

## Methods

### Cell culture and membrane preparation

CHO cells were grown to confluence in 75 cm^2^ flasks in Dulbecco’s modified Eagle’s medium (DMEM) supplemented with 10% fetal bovine serum. Two million cells were subcultured in 100 mm Petri dishes. The medium was supplemented with 5 mM sodium butyrate for the last 24 h of cultivation to increase receptor expression. Cells were washed with phosphate-buffered saline and manually harvested on day 5 after subculture and centrifuged for 3 min at 250 × g. The pellet was suspended in 10 ml of ice-cold homogenization medium (100 mM NaCl, 20 mM Na-HEPES, 10 mM EDTA, pH = 7.4) and homogenized on ice by two 30 s strokes using a Polytron homogenizer (Ultra-Turrax; Janke & Kunkel GmbH & Co. KG, IKA-Labortechnik, Staufen, Germany) with a 30-s pause between strokes. Cell homogenates were centrifuged for 5 min at 1,000 × g. The supernatant was collected and centrifuged for 30 min at 30,000 × g. Pellets were suspended in the washing medium (100 mM, 10 mM MgCl_2_, 20 mM Na-HEPES, pH = 7.4), left for 30 min at 4 °C, and then centrifuged again for 30 min at 30,000 × g. Resulting membrane pellets were kept at -80 °C until assayed.

### Radioligand binding experiments

All radioligand binding experiments were optimized and carried out according to general guidelines^33^. Membranes (20 to 50 μg of membrane proteins per sample) were incubated in 96-well plates for 3 h at 37 °C in 400 μl of Krebs-HEPES buffer (KHB; final concentrations in mM: NaCl 138; KCl 4; CaCl_2_ 1.3; MgCl_2_ 1; NaH_2_PO_4_ 1.2; HEPES 20; glucose 10; pH adjusted to 7.4). In saturation experiments of binding of [^3^H]N-methylscopolamine ([^3^H]NMS) six concentrations of the radioligand (ranging from 63 to 2000 pM) were used. Agonist binding was determined in competition experiments with 1 nM [^3^H]NMS. Nonspecific binding was determined in the presence of 10 μM atropine. Incubation was terminated by filtration through Whatman GF/C glass fibre filters (Whatman) using a Brandel harvester (Brandel, USA). Filters were dried in a microwave oven (3 min, 800 W) and then solid scintillator Meltilex A was melted on filters (105 °C, 60 s) using a hot plate. The filters were cooled and counted in a Wallac Microbeta scintillation counter (Wallac, Finland).

### Accumulation of Inositol phosphates

Accumulation of inositol phosphates (IP_X_) was assayed in cells in suspension. IP_X_ was determined after separation on ion-exchange columns (Dowex 1X8-200, Sigma, USA). Cells were harvested by mild trypsinization and resuspended in KHB and centrifuged 250 g for 3 min. Cells were resuspended in KHB supplemented with 500 nM [^3^H]myo-inositol (ARC, USA) and incubated at 37 °C for 1 h. Then they were washed once with an excess of KHB, resuspended in KHB containing 10 mM LiCl, and incubated for 1 h at 37 °C in the presence of indicated concentrations of agonists and/or allosteric modulator. Incubation was terminated by the addition of 0.5 ml of stopping solution (chloroform: methanol: HCl; 2: 1: 0.1) and placed in 4 °C for 1 h. An aliquot (0.6 ml) of the upper (aqueous) phase was taken and loaded onto ion-exchange columns. Columns were washed with 10 ml of deionized water and 20 ml of 60 mM ammonium formate/5 mM sodium borate solution. IP_X_ were collectively eluted from columns by 4 ml of 1 M ammonium formate-0.1 M/formic acid buffer.

### Analysis of experimental data

Data from experiments were processed in Libre Office and then analysed and plotted using program Grace (https://plasma-gate.weizmann.ac.il/Grace). The following equations were used for non-linear regression analysis:

26 $$\begin{aligned} & \left[ {^{3} {\text{H}}} \right]{\text{NMS}}\,{\text{saturation}}\,{\text{binding}} \\ & y = \frac{{B_{{{\text{MAX}}}} *x}}{{{\text{x } + \text{ K}}_{D} }} \\ \end{aligned}$$

where y is specific binding at free concentration x, B_MAX_ is the maximum binding capacity, and K_D_ is the equilibrium dissociation constant.

27 $$\begin{aligned} & {\text{Competition}}\,{\text{binding}} \\ & y = {100} - \left( {{100} - f_{{{\text{low}}}} } \right)*\frac{x}{{{\text{x } + \text{ IC}}_{{{\text{50high}}}} }} - f_{{{\text{low}}}} *\frac{x}{{{\text{x } + \text{ IC}}_{{{\text{50low}}}} }} \\ \end{aligned}$$

where y is specific radioligand binding at concentration x of competitor expressed as per cent of binding in the absence of a competitor, IC_50_ is the concentration causing 50% inhibition of radioligand binding at high (IC_50high_) and low (IC_50low_) affinity binding sites, f_low_ is the fraction of low-affinity binding sites expressed in per cent. Inhibition constant K_I_ was calculated as:

28 $$K_{I} = \frac{{{\text{IC}}_{{{50}}} }}{{1 + \frac{\left[ D \right]}{{K_{D} }}}}$$

where [D] is the concentration of radioligand used and K_D_ is its equilibrium dissociation constant.

### Allosteric interaction

Interaction between tracer ([^3^H]NMS) and allosteric modulator:

29 $$y = \frac{{\left[ D \right] + K_{D} }}{{\left[ D \right] + K_{D} \frac{{K_{B} + x}}{{K_{B} + x \alpha }}}}$$

where y is specific radioligand binding at concentration x of the allosteric modulator as per cent of binding in the absence of allosteric modulator. Where [D] and K_D_ are concentration and equilibrium dissociation constant of the tracer ([^3^H]NMS). The equilibrium dissociation constant of the allosteric modulator K_B_ and factor of binding cooperativity α are obtained by fitting of Eq. (29) to data.

Interaction between tracer ([^3^H]NMS) and allosteric modulator in the presence of an agonist at fixed concentration:

30 $$y = \frac{{\left[ D \right] + K_{D} }}{{\left[ D \right] + K_{D} \frac{{\left[ A \right]\left( {K_{B} + \left[ B \right] \alpha_{2} } \right) + K_{A} \left( {K_{B} + \left[ B \right]} \right)}}{{K_{A} \left( {K_{B} + \left[ B \right] \alpha_{1} } \right)}}}}$$

where y is specific radioligand binding at concentration x of the allosteric modulator as per cent of binding in the absence of allosteric modulator. Where [D] and K_D_ are concentration and equilibrium dissociation constant of the tracer ([^3^H]NMS), [A] and K_A_ are concentration and equilibrium dissociation constant of the agonist. Parameters K_B_ and α_1_ obtained by fitting of Eq. (29) to binding data in the absence of the agonist.

31 $$\begin{aligned} & {\text{Concentration } - \text{ response}}\,{\text{curve}} \\ & y = basal + \frac{{\left( {{\text{E}}_{{{\text{MAX}}}}^{\prime } - basal} \right)*x^{{{\text{nH}}}} }}{{x^{{{\text{nH}}}} {\text{ } + \text{ EC}}_{{{50}}}^{{{\text{nH}}}} }} \\ \end{aligned}$$

where y is response normalized to basal activity in the absence of allosteric ligand at concentration x, E'_MAX_ is the apparent maximal response, EC_50_ is concentration causing half-maximal effect, and n_H_ is Hill coefficient.

## Supplementary information

Supplementary Information
