# Supplementary material for: The operational model of allosteric modulation of pharmacological agonism
Source: Sci Rep. 2020 Sep 2;10:14421. doi: 10.1038/s41598-020-71228-y (PMC7468285; doi:10.1038/s41598-020-71228-y)
Supplement: Supplementary file 1 — Supplementary Information [file 41598_2020_71228_MOESM1_ESM.pdf]

Supplementary information: Operational model of allosteric modulation of pharmacological agonism

Jan Jakubík[1], Alena Randáková[1], Nikolai Chetverikov[1], Esam E. El-Fakahany[2], and Vladimír Doležal[1],

[1], Institute of Physiology CAS, 142 20 Prague, Czech Republic.

[2], Department of Experimental and Clinical Pharmacology, University of Minnesota College of Pharmacy, Minneapolis, MN 55455, USA.

Contents

Supplementary Figures 2
- Figure S1 Effects of a negative allosteric modulator on the functional response to an orthosteric agonist 2
- Figure S2 Effects of a positive allosteric modulator on the functional response to an orthosteric agonist 3
- Figure S3 Effects of a negative allosteric agonist on the functional response to orthosteric agonist 4
- Figure S4 Effects of a positive allosteric agonist on the functional response to an orthosteric agonist 5

Supplementary Tables 6
- Table S1 Binding parameters of agonists 6
- Table S2 Binding parameters of allosteric ligands 6
- Table S3 Parameters of functional response to orthosteric and allosteric agonists 6
- Table S4 Parameters of allosteric modulation of functional response to carbachol 7
- Table S5 Parameters of allosteric modulation of functional response to iperoxo 7

Derivation of equations 8
- Equations of the operational model of agonism 8
- Equations of the operational model of allosteric modulation of agonism 8
  - Full model 8
  - For β=1 9
  - For α=1 10
- Equations of operational model of allosteric agonist 10
  - Full model 10
  - For α=1 and β=1 11
  - For β=1 12
  - For α=1 12

Supplementary Figures

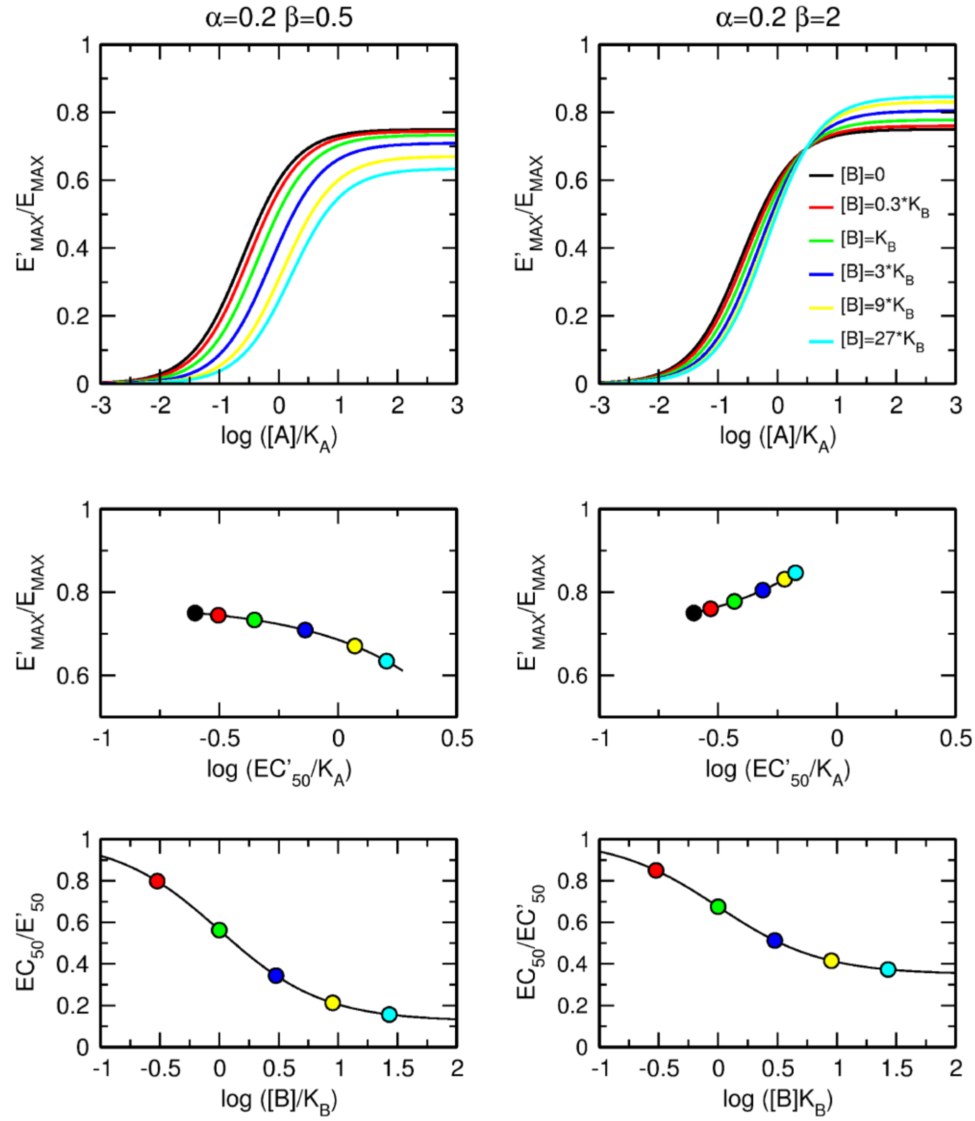

Figure S1 Effects of a negative allosteric modulator on the functional response to an orthosteric agonist

Effects of a negative allosteric modulator exerting negative (left) or positive (right) operational cooperativity on the functional response to an orthosteric agonist (top), observed half-efficient concentration EC'_{50} and observed maximal response E'_{MAX} (middle), and dose ratio of half-efficient concentrations (bottom). $E_{MAX} = 1$, $\tau_A = 3$, values of factors of cooperativity α and β are indicated within the plots.

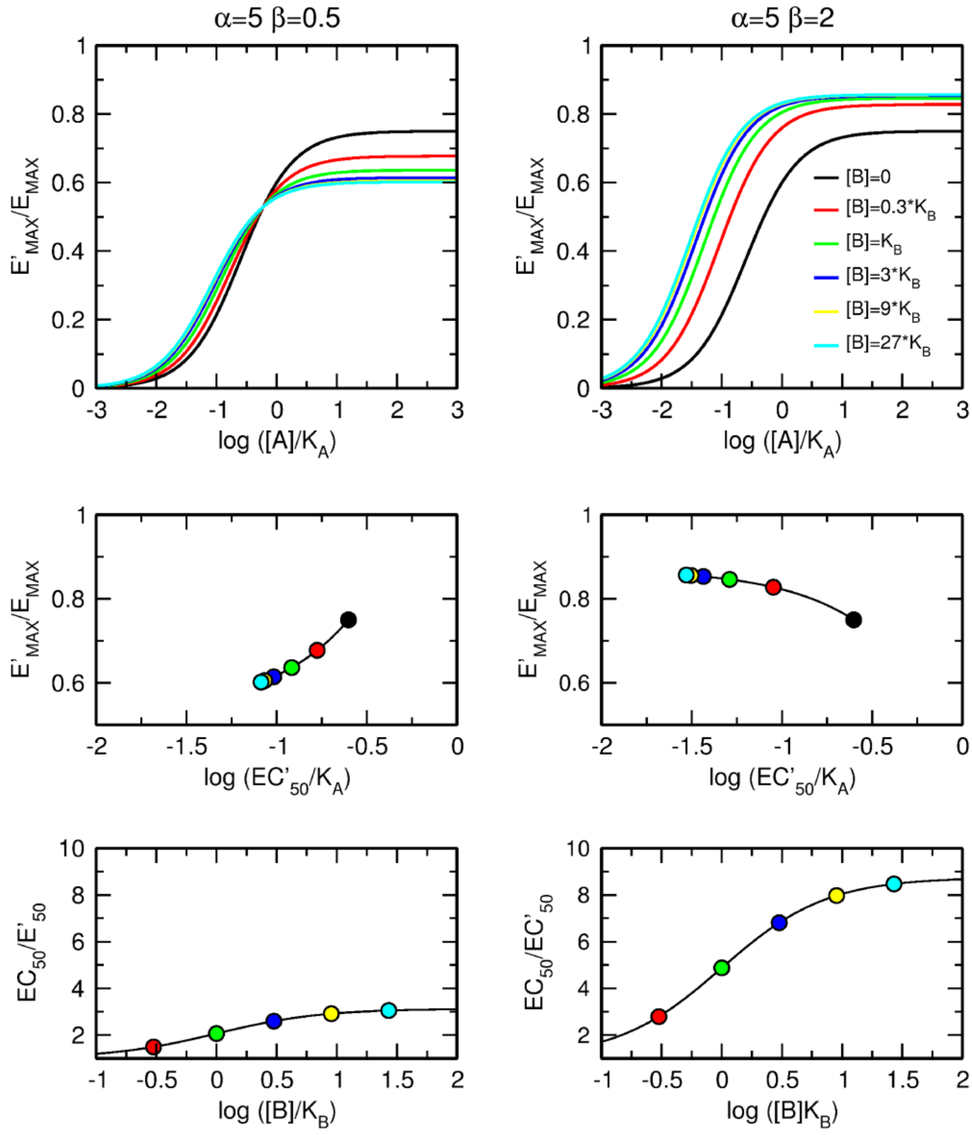

Figure S2 Effects of a positive allosteric modulator on the functional response to an orthosteric agonist

Effects of a positive allosteric modulator exerting negative (left) or positive (right) operational cooperativity on functional response to an orthosteric agonist (top), observed half-efficient concentration EC'_{50} and observed maximal response E'_{MAX} (middle), and dose ratio of half-efficient concentrations (bottom). $E_{MAX} = 1$, $\tau_A = 3$, values of factors of cooperativity α and β are indicated within the plots.

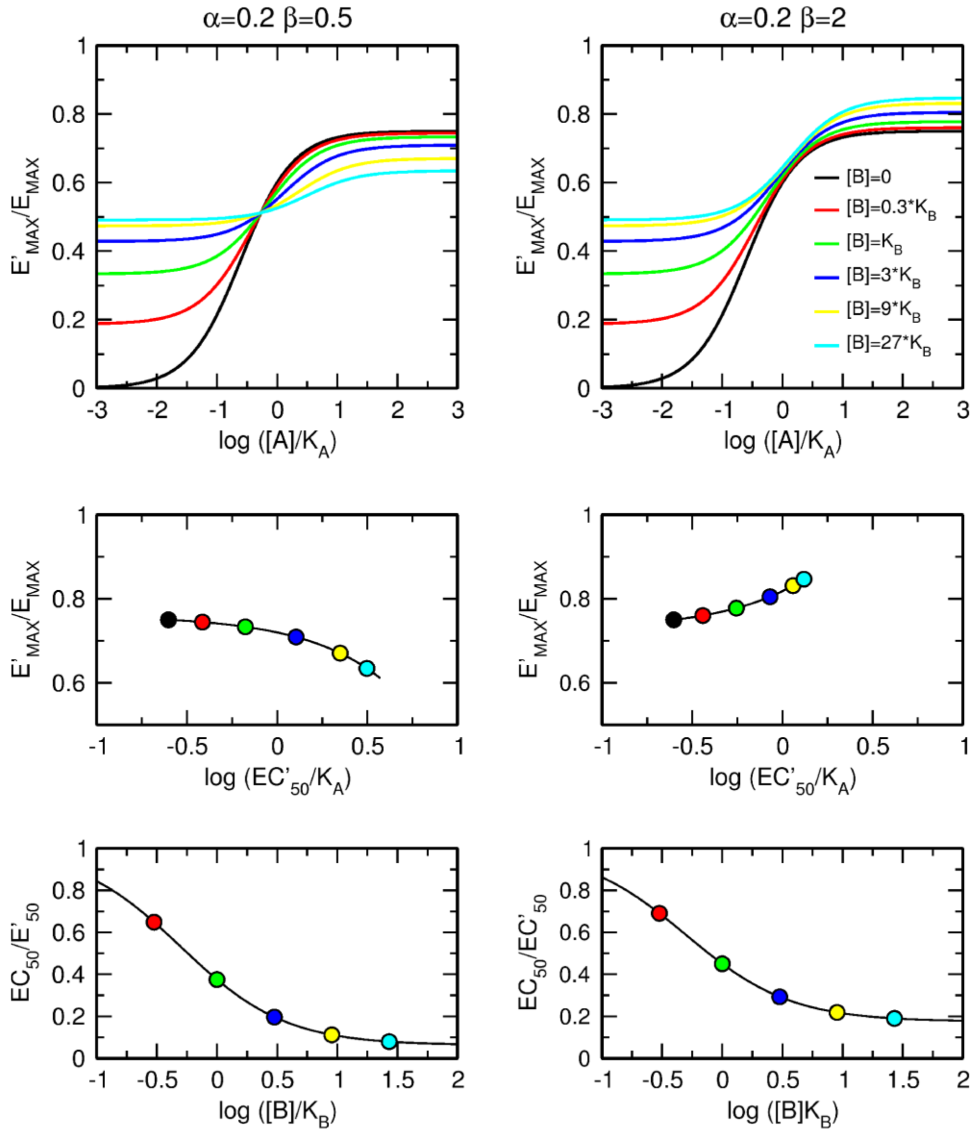

Figure S3 Effects of a negative allosteric agonist on the functional response to orthosteric agonist

Effects of allosteric agonist, exerting negative binding cooperativity and negative (left) or positive (right) operational cooperativity on the functional response to an orthosteric agonist (top), observed half-efficient concentration EC'_{50} and observed maximal response E'_{MAX} (middle), and dose ratio of half-efficient concentrations (bottom). $E_{MAX} = 1$, $\tau_A = 3$, $\tau_B = 1$, values of factors of cooperativity α and β are indicated within the plots.

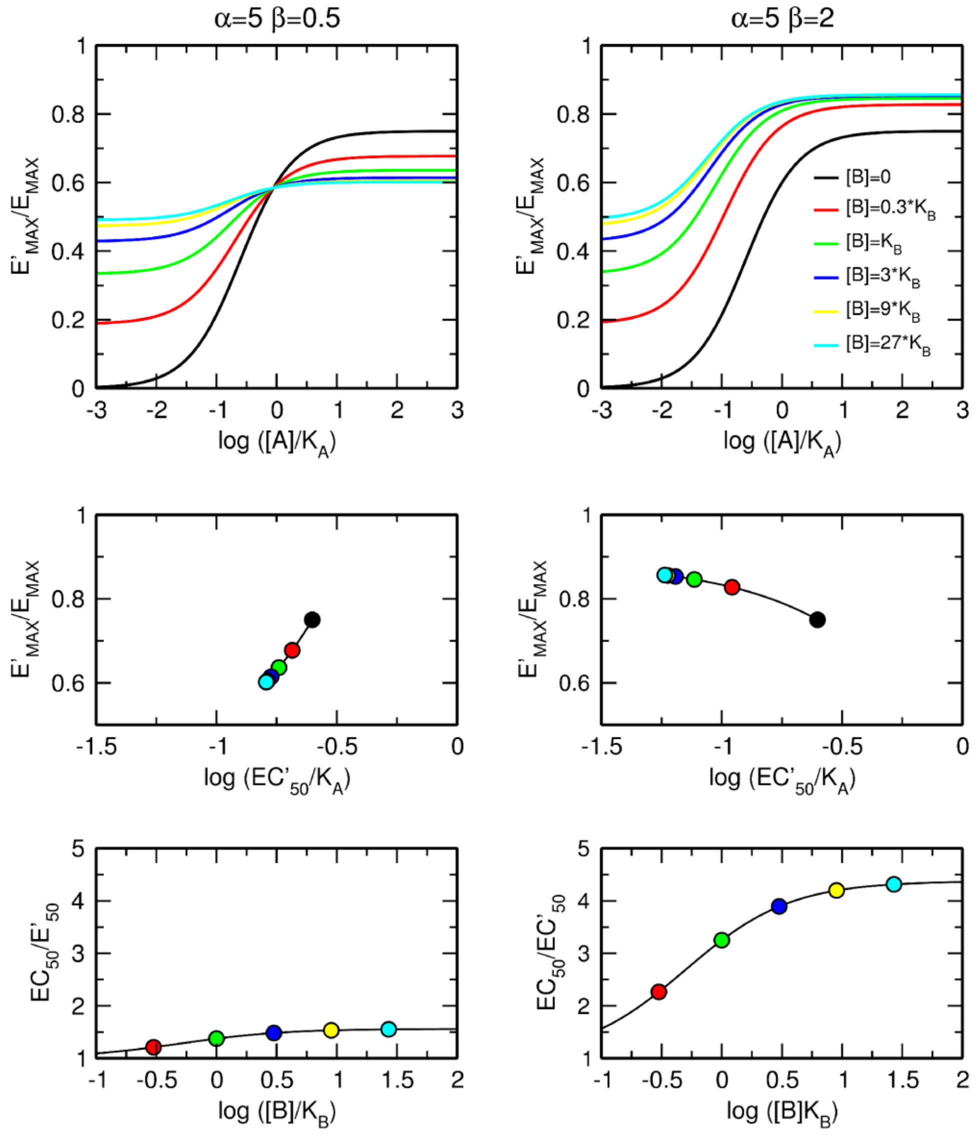

Figure S4 Effects of a positive allosteric agonist on the functional response to an orthosteric agonist

Effects of an allosteric agonist exerting positive binding cooperativity and negative (left) or positive (right) operational cooperativity on functional response to orthosteric agonist (top), observed half-efficient concentration EC'_{50} and observed maximal response E'_{MAX} (middle), and dose ratio of half-efficient concentrations (bottom). $E_{MAX} = 1$, $\tau_A = 3$, $\tau_B = 1$, values of factors of cooperativity α and β are indicated within the plots.

Supplementary Tables

Table S1 Binding parameters of agonists

Agonist half-efficient concentrations IC_{50} and fractions of low-affinity sites were obtained by fitting equation (27) to the data in Figure 11. Equilibrium dissociation constants K_I were calculated according to equation (28). Values of IC_{50} and K_I are expressed as negative logarithms. Values are estimates ± SD.

	pIC_{50high}	pIC_{50low}	flow [%]	pK_{Ihigh}	pK_{Ilow}
carbachol	5.71 ± 0.05	4.20 ± 0.08	72 ± 9	6.41 ± 0.05	4.90 ± 0.08
iperoxo	8.99 ± 0.05	5.96 ± 0.04	51 ± 5	9.70 ± 0.05	6.66 ± 0.04

Table S2 Binding parameters of allosteric ligands

Equilibrium dissociation constant K_B of allosteric ligands and their binding cooperativity with NMS was obtained by fitting equation (29) to the data in Figure 12. Binding cooperativities between allosteric ligands and agonist were obtained by fitting equation (30) to the data in Figure 12. Values of K_B are expressed as negative logarithms. Values are estimates ± SD.

	pK_B	α_{NMS}	$\alpha_{carbachol}$	$\alpha_{iperoxo}$
BQCA	4.32 ± 0.06	0.29 ± 0.00	33 ± 3	4.8 ± 0.2
TBPB	6.30 ± 0.05	0.002 ± 0.001	0.7 ± 0.3	0.8 ± 0.4

Table S3 Parameters of functional response to orthosteric and allosteric agonists

Half-efficient concentrations EC_{50} and apparent maximal responses E'_{MAX} were obtained by fitting equation (31) to the data in Figure 13. Operational efficacy τ_A and equilibrium dissociation constant K_A were obtained by fitting equation (1) to the data in Figure 13. System maximal response E_{MAX} was either fixed to 98 (A) or estimated as a shared parameter of all 4 curves (B). Values are estimates ± SD.

**A**	pEC_{50}	E'_{MAX}	n_H	τ_A	pK_A
BQCA	7.05 ± 0.02	34.6 ± 0.4	1.01 ± 0.06	0.52 ± 0.01	6.87 ± 0.03
carbachol	6.20 ± 0.04	61.0 ± 1.3	1.05 ± 0.07	1.64 ± 0.07	5.75 ± 0.04
iperoxo	9.42 ± 0.01	64.7 ± 0.5	1.04 ± 0.02	1.86 ± 0.04	8.96 ± 0.02
TBPB	7.39 ± 0.04	54.4 ± 0.9	1.01 ± 0.08	1.20 ± 0.04	7.05 ± 0.04

**B**	E_{MAX}	τ_A	pK_A
BQCA	98 ± 53	0.56 ± 695	6.88 ± 193
carbachol	98 ± 53	1.78 ± 417	5.76 ± 646
iperoxo	98 ± 53	1.97 ± 708	8.96 ± 104
TBPB	98 ± 53	1.26 ± 334	6.95 ± 331

Table S4 Parameters of allosteric modulation of functional response to carbachol

A; Half-efficient concentrations EC_{50} and apparent maximal responses E'_{MAX} were obtained by fitting equation (31) to the data in Figure 14. B; Cooperativity factors α and β were obtained as shared parameters of all 3 curves of fitting equation (14) to the data in Figure 14. Operational efficacies τ_A and τ_B and equilibrium dissociation constants K_A and K_A and maximal system response E_{MAX} were fixed to values in Table S3. Initial guess values of β and α were calculated according to equation (8) and (15), respectively. Values are estimates ± SD.

**A**	**pEC50**	**E'MAX**	**nH**	**basal**	**EC50/EC'50**	**E'MAX/EMAX**
Carbachol	6.20 ± 0.04	61.0 ± 1.3	1.05 ± 0.07	1		
+ 30 µM BQCA	7.54 ± 0.06	67.2 ± 0.8	0.92 ± 0.13	34.4	21.88	0.682
+ 100 µM BQCA	7.58 ± 0.06	67.9 ± 1.0	0.91 ± 0.10	34.5	23.99	0.689
+ 300 µM BQCA	7.60 ± 0.06	68.3 ± 1.5	0.90 ± 0.15	34.5	25.12	0.693
+ 1 µM TBPB	6.16 ± 0.24	57.8 ± 3.3	1.18 ± 0.20	51.3	0.912	0.586
+ 3 µM TBPB	6.00 ± 0.28	57.9 ± 1.4	0.85 ± 0.15	52.7	0.631	0.586
+ 10 µM TBPB	6.01 ± 0.33	56.0 ± 2.2	1.15 ± 0.18	53.2	0.645	0.567

**B**	**α**	**β**
BQCA	28 ± 3	1.25 ± 0.08
TBPB	0.48 ± 0.05	0.80 ± 0.05

Table S5 Parameters of allosteric modulation of functional response to iperoxo

A; Half-efficient concentrations EC_{50} and apparent maximal responses E'_{MAX} were obtained by fitting equation (31) to the data in Figure 14. B; Cooperativity factors α and β were obtained as shared parameters of all 3 curves of fitting equation (14) to the data in Figure 14. Operational efficacies τ_A and τ_B and equilibrium dissociation constants K_A and K_A and maximal system response E_{MAX} were fixed to values in Table S3. Initial guess values of β and α were calculated according to equation (8) and (15), respectively. Values are estimates ± SD.

**A**	**pEC50**	**E'MAX**	**nH**	**basal**	**EC50/EC'50**	**E'MAX/EMAX**
Iperoxo	9.42 ± 0.01	64.7 ± 0.5	1.04 ± 0.02	1		
+ 30 µM BQCA	10.22 ± 0.06	76.3 ± 1.3	0.97 ± 0.12	34.4	6.310	0.776
+ 100 µM BQCA	10.23 ± 0.06	76.2 ± 1.3	1.09 ± 0.15	34.5	6.457	0.775
+ 300 µM BQCA	10.25 ± 0.09	76.6 ± 1.8	0.95 ± 0.16	34.5	6.761	0.779
+ 1 µM TBPB	9.07 ± 0.41	59.7 ± 3.9	1.18 ± 0.20	51.3	0.446	0.605
+ 3 µM TBPB	8.90 ± 0.85	58.0 ± 4.8	0.82 ± 0.20	52.7	0.302	0.588
+ 10 µM TBPB	8.93 ± 0.50	57.6 ± 2.9	1.19 ± 0.22	53.2	0.324	0.583

**B**	**α**	**β**
BQCA	6.2 ± 0.8	1.8 ± 0.2
TBPB	0.51 ± 0.08	0.78 ± 0.08

Derivation of equations

Equations of the operational model of agonism

According to the operational model (OM) of agonism, a functional response (Resp) is given by Eq. 1

$$Response = \frac{[A]\tau_A E_{MAX}}{[A](\tau_A+1)+K_A} \qquad \text{(Eq. 1)}$$

Where [A] is the concentration of an agonist, E_{MAX} is the maximal response of the system, K_A is the equilibrium dissociation constant of the agonist-receptor complex and τ is the operational factor of efficacy. According to the OM, EC_{50} is related to K_A according to the following equation.

$$EC_{50} = \frac{K_A}{\tau_A+1} \qquad \text{(Eq. 2)}$$

The apparent maximal response E'_{MAX} is measured as the upper asymptote of the functional response curve described in Eq. 3.

$$E'_{MAX} = \frac{\tau_A E_{MAX}}{\tau_A+1} \qquad \text{(Eq. 3)}$$

By rearrangement of Eq. 2, τ can be expressed as follows.

$$\tau_A = \frac{K_A-EC_{50}}{EC_{50}} \qquad \text{(Eq. 4)}$$

After substitution of τ in Eq. 3, its rearrangement and simplification we get the relation between observed parameters E'_{MAX} and EC_{50} and objective parameters E_{MAX} and K_A according to Eq. 5.

$$E'_{MAX} = E_{MAX} - \frac{E_{MAX}EC_{50}}{K_A} \qquad \text{(Eq. 5)}$$

Equations of the operational model of allosteric modulation of agonism

Full model

Allosterically-modulated response to an agonist follows the operational model given by Eq. 6.

$$Response = \frac{E_{MAX}\tau_A[A](K_B+\alpha\beta[B])}{[A]K_B+K_AK_B+[B]K_A+\alpha[A][B]+\tau_A[A](K_B+\alpha\beta[B])} \qquad \text{(Eq. 6)}$$

Where [A] and [B] are the concentrations of an agonist and allosteric modulator, respectively, E_{MAX} is the maximal response of the system, K_A and K_B are the equilibrium dissociation constants of the agonist-receptor and allosteric modulator-receptor complex, respectively, and τ_A is the operational factor of efficacy of an agonist.

Let’s define δ

$$\delta = K_B + \alpha\beta[B] \qquad \text{(Eq. 7)}$$

Then Eq. 6 becomes Eq. 8.

$$Response = \frac{\delta\tau_A E_{MAX}[A]}{[A]K_B+K_AK_B+[B]K_A+\alpha[A][B]+\delta\tau_A[A]} \qquad \text{(Eq. 8)}$$

Simplification of Eq. 8 gives Eq. 9

$$Response = \frac{\delta\tau_A E_{MAX}[A]}{[A](\alpha[B]+\delta\tau_A+K_B)+K_A([B]+K_B)} \qquad \text{(Eq. 9)}$$

From Eq. 9 the parameters of the concentration-response curve, the observed maximal response E'_{MAX} to an agonist A, its half-efficient concentration EC'_{50} and dose ratio are given by Eq. 10 and 11, respectively:

$$E'_{MAX} = \frac{\delta\tau_A E_{MAX}}{\alpha[B]+\delta\tau_A+K_B} \quad \text{(Eq. 10)}$$

$$EC'_{50} = \frac{K_A([B]+K_B)}{\alpha[B]+\delta\tau_A+K_B} \quad \text{(Eq. 11)}$$

The ratio of the half-efficient concentration of an agonist in the absence of an allosteric modulator EC_{50} to its presence at concentration [B] (where values >1 denote positive cooperativity and values <1 denote negative cooperativity) is given by Eq. 12.

$$\frac{EC_{50}}{EC'_{50}} = \frac{\alpha[B]+(K_B+\alpha\beta[B])\tau_A+K_B}{(\tau_A+1)(K_B+[B])} \quad \text{(Eq. 12)}$$

From Eq. 11 K_A and τ_A can be expressed as Eq. 13 and 14, respectively.

$$K_A = \frac{EC'_{50}(\alpha[B]+\delta\tau_A+K_B)}{[B]+K_B} \quad \text{(Eq. 13)}$$

$$\tau_A = \frac{K_A[B]+K_AK_B-\alpha[B]EC'_{50}-K_BEC'_{50}}{\delta EC'_{50}} \quad \text{(Eq. 14)}$$

Substitution of τ_A in Eq. 10 gives Eq. 15.

$$E'_{MAX} = \frac{E_{MAX}(K_A(K_B+[B])-\alpha[B]EC'_{50}-K_BEC'_{50})}{K_A(K_B+[B])} \quad \text{(Eq. 15)}$$

Rearrangement of Eq. 10 to express τ_A gives Eq. 16.

$$\tau_A = E'_{MAX}\frac{K_B+\alpha[B]}{\delta(E_{MAX}-E'_{MAX})} \quad \text{(Eq. 16)}$$

Substitution of τ_A in Eq. 11 gives Eq. 17.

$$EC'_{50} = \frac{K_A(K_B+[B])(E_{MAX}-E'_{MAX})}{E_{MAX}(K_B+\alpha[B])} \quad \text{(Eq. 17)}$$

And dose ratio is given by the division of Eq. 2 by Eq. 17:

$$\frac{EC_{50}}{EC'_{50}} = \frac{E_{MAX}(K_B+\alpha[B])}{(\tau+1)(K_B+[B])(E_{MAX}-E'_{MAX})} \quad \text{(Eq. 18)}$$

For β=1

For the factor of operational cooperativity β=1, equations for E'_{MAX}, and EC'_{50} can be simplified. E'_{MAX} is given by Eq. 19.

$$E'_{MAX} = \frac{(K_B+[B])\tau_A E_{MAX}}{\alpha[B]+(K_B+\alpha[B])\tau_A+K_B} \quad \text{(Eq. 19)}$$

After simplification, Eq. 19 becomes Eq. 20.

$$E'_{MAX} = \frac{E_{MAX}\tau_A}{\tau_A+1} \quad \text{(Eq. 20)}$$

Eq. 20 means that for β=1 E'_{MAX} value is constant.

Eq. 11 in expanded form for β=1 is Eq. 21.

$$EC'_{50} = \frac{K_A([B]+K_B)}{\alpha[B]+(K_B+\alpha[B])\tau_A+K_B} \quad \text{(Eq. 21)}$$

Division of Eq. 2 by Eq. 21 gives the dose ratio:

$$\frac{EC_{50}}{EC'_{50}} = \frac{\alpha[B]+(K_B+\alpha[B])\tau_A+K_B}{(\tau_A+1)(K_B+[B])} \qquad \text{(Eq. 22)}$$

After simplification, Eq. 22 becomes Eq. 23.

$$\frac{EC_{50}}{EC'_{50}} = \frac{K_B+\alpha[B]}{K_B+[B]} \qquad \text{(Eq. 23)}$$

For α=1

For a factor of binding cooperativity α=1 equations for E'_{MAX}, and EC'_{50} can be simplified. E'_{MAX} is given by Eq. 24 and τ_A is given by Eq. 25.

$$E'_{MAX} = \frac{(K_B+\beta[B])\tau_A E_{MAX}}{[B]+(K_B+\beta[B])\tau_A+K_B} \qquad \text{(Eq. 24)}$$

$$\tau_A = \frac{K_A[B]+K_AK_B-[B]EC'_{50}-K_BEC'_{50}}{(K_B+\beta[B])EC'_{50}} \qquad \text{(Eq. 25)}$$

After substitution of τ_A (Eq. 25) in Eq. 24 and simplification E'_{MAX} is given by Eq. 26.

$$E'_{MAX} = E_{MAX}\left(1-\frac{EC'_{50}}{K_A}\right) \qquad \text{(Eq. 26)}$$

For α=1 Eq. 11 in expanded form becomes Eq. 27.

$$EC'_{50} = \frac{K_A(K_B+[B])}{[B]\tau_A(K_B+\beta[B])+K_B} \qquad \text{(Eq. 27)}$$

Division of Eq. 2 by Eq. 27 gives the dose ratio:

$$\frac{EC_{50}}{EC'_{50}} = \frac{[B]\tau_A(K_B+\beta[B])+K_B}{(K_B+[B])(\tau_A+1)} \qquad \text{(Eq. 28)}$$

After rearrangement

$$\frac{EC_{50}}{EC'_{50}} = \frac{\tau_A(\beta[B]+K_B)+[B]+K_B}{\tau_A([B]+K_B)+[B]+K_B} \qquad \text{(Eq. 29)}$$

Equations of the operational model of allosteric agonist

An allosteric agonist is an allosteric modulator that elicits a functional response on its own with intrinsic activity τ_B. Response to the allosteric agonist B alone is given by Eq. 30.

$$Response = \frac{[B]\tau_B E_{MAX}}{[B](\tau_B+1)+K_B} \qquad \text{(Eq. 30)}$$

Full model

Then the response to an agonist A in the presence of an allosteric agonist B is given by Eq. 31.

$$Response = \frac{E_{MAX}(\tau_A[A](K_B+\alpha\beta[B])+\tau_B[B]K_A)}{[A]K_B+K_AK_B+[B]K_A+\alpha[A][B]+\tau_A[A](K_B+\alpha\beta[B])+\tau_B[B]K_A} \qquad \text{(Eq. 31)}$$

Let's define φ

$$\varphi = \tau_B[B]K_A \qquad \text{(Eq. 32)}$$

Using δ and φ, Eq. 31 becomes Eq. 33.

$$Response = \frac{E_{MAX}(\tau_A[A]\delta+\varphi)}{[A]K_B+K_AK_B+[B]K_A+\alpha[A][B]+\tau_A[A]\delta+\varphi} \qquad \text{(Eq. 33)}$$

Rearrangement of Eq. 33 gives Eq. 34.

$$Response = \frac{E_{MAX}(\tau_A[A]\delta+\varphi)}{[A](\alpha[B]+\delta\tau_A+K_B)+K_A([B]+K_B)+\varphi} \quad \text{(Eq. 34)}$$

From Eq. 34 the parameters of the concentration-response curve, the observed half-efficient concentration EC'_{50} and observed maximal response E'_{MAX} to an agonist A are given by Eq. 35 and 36, respectively:

$$E'_{MAX} = \frac{\delta\tau_A E_{MAX}}{\alpha[B]+\delta\tau_A+K_B} \quad \text{(Eq. 35)}$$

$$EC'_{50} = \frac{K_A([B]+K_B)+\varphi}{\alpha[B]+\delta\tau_A+K_B} \quad \text{(Eq. 36)}$$

Division of Eq. 2 by Eq. 36 gives the dose ratio:

$$\frac{EC_{50}}{EC'_{50}} = \frac{\alpha\beta[B]\tau_A+\alpha[B]+K_B\tau_A+K_B}{(\tau_A+1)([B]\tau_B+[B]+K_B)} \quad \text{(Eq. 37)}$$

Eq. 35 is the same as Eq. 10 thus Eq. 16 can be used to express τ_A and substitute it in Eq. 36 to get Eq. 38.

$$EC'_{50} = \frac{K_A([B]+K_B)+[B]K_A\tau_B}{\alpha[B]+\frac{E'_{MAX}(\alpha[B]+K_B)}{E_{MAX}-E'_{MAX}}+K_B} \quad \text{(Eq. 38)}$$

Division of Eq. 2 by Eq. 38 gives the dose ratio:

$$\frac{EC_{50}}{EC'_{50}} = \frac{E_{MAX}(\alpha[B]+K_B)}{(\tau_A+1)(E_{MAX}-E'_{MAX})([B]\tau_B+[B]+K_B)} \quad \text{(Eq. 39)}$$

By rearrangement of Eq. 36 τ_A can be expressed as

$$\tau_A = \frac{\frac{K_A([B]+K_B+[B]\tau_B)}{EC'_{50}}-\alpha[B]-K_B}{K_B+\alpha\beta[B]} \quad \text{(Eq. 40)}$$

After substitution of τ_A in Eq. 35 by Eq. 40

$$E'_{MAX} = EC'_{50}E_{MAX}\frac{\frac{K_A([B]\tau_B+[B]+K_B)}{EC'_{50}}-\alpha[B]-K_B}{K_A([B]\tau_B+[B]+K_B)} \quad \text{(Eq. 41)}$$

After simplification, Eq. 41 becomes Eq. 42

$$E'_{MAX} = E_{MAX}\frac{[B](K_A\tau_B+K_A-\alpha EC'_{50})+K_B(K_A-EC'_{50})}{K_A([B]\tau_B+[B]+K_B)} \quad \text{(Eq. 42)}$$

For α=1 and β=1

For α=1 and β=1, E'_{MAX} value is given by Eq. 43:

$$E'_{MAX} = \frac{(K_B+[B])\tau_A E_{MAX}}{[B]+(K_B+[B])\tau_A+K_B} \quad \text{(Eq. 43)}$$

After simplification, Eq. 43 becomes Eq. 44.

$$E'_{MAX} = \frac{\tau_A E_{MAX}}{\tau_A+1} \quad \text{(Eq. 44)}$$

Eq. 44 means that for α=1 and β=1 E'_{MAX} value is constant (independent from allosteric agonist B) and is given by operational efficacy of an orthosteric agonist τ_A.

For α=1 and β=1, E'_{50} value is given by Eq. 45:

$$EC'_{50} = \frac{K_A([B]+K_B)+\tau_B[B]K_A}{[B]+(K_B+[B])\tau_A+K_B}$$ (Eq. 45)

After rearrangement, Eq. 45 becomes Eq. 46

$$EC'_{50} = \frac{K_A([B]\tau_B+[B]+K_B)}{(\tau_A+1)([B]+K_B)}$$ (Eq. 46)

Division of Eq. 2 by Eq. 46 gives the dose ratio:

Eq. 47 $$\frac{EC_{50}}{EC'_{50}} = \frac{[B]+K_B}{[B]\tau_B+[B]+K_B}$$

For β=1

For β=1, E'_{MAX} value is given by Eq. 48

$$E'_{MAX} = \frac{(K_B+[B])\tau_A E_{MAX}}{\alpha[B]+(K_B+\alpha[B])\tau_A+K_B}$$ (Eq. 48)

After simplification, Eq. 49 becomes Eq. 49.

$$E'_{MAX} = \frac{E_{MAX}\tau_A}{\tau_A+1}$$ (Eq. 49)

Eq. 49 means that for β=1 E'_{MAX} value is constant (independent from allosteric agonist B) is given by operational efficacy of an orthosteric agonist τ_A.

For β=1, EC'_{50} value is given by Eq. 50.

$$EC'_{50} = \frac{K_A([B]+K_B)+\tau_B[B]K_B}{\alpha[B]+(K_B+\alpha[B])\tau_A+K_B}$$ (Eq. 50)

Then the division of Eq. 2 by Eq. 50 gives dose ratio:

$$\frac{EC_{50}}{EC'_{50}} = \frac{K_A(\alpha[B]+(K_B+\alpha[B])\tau_A+K_B)}{(\tau_A+1)(K_A([B]+K_B)+\tau_B[B]K_B)}$$ (Eq. 51)

After substitution of τ_A and simplification Eq. 51 becomes Eq 52.

$$\frac{EC_{50}}{EC'_{50}} = \frac{K_A(K_B+\alpha[B])}{K_B[B]+K_AK_B+\tau_B[B]K_B}$$ (Eq. 52)

After rearrangement, Eq. 52 becomes Eq. 53.

$$\frac{EC_{50}}{EC'_{50}} = \frac{\alpha[B]+K_B}{[B]\tau_B+[B]+K_B}$$ (Eq. 53)

For α=1

As Eq. 35 is the same as Eq. 10 for allosteric agonist with α=1 observed maximal response E'_{MAX} is given by Eq. 26. Eq. 54 describes the observed half-efficient concentration of an agonist EC'50.

$$EC'_{50} = \frac{K_A([B]+K_B)+\tau_B[B]K_A}{[B]+(\beta[B]+K_B)\tau_A+K_B}$$ (Eq. 54)

After rearrangement:

$$EC'_{50} = \frac{K_A([B]+K_B+\tau_B[B])}{[B]+(\beta[B]+K_B)\tau_A+K_B}$$ (Eq. 55)

The dose ratio is:

$$\frac{EC_{50}}{EC'_{50}} = \frac{[B]+\beta[B]\tau_A+K_B\tau_A+K_B}{(\tau_A+1)([B]+K_B+\tau_B[B])}$$ (Eq. 56)
